# Identification of Biologically Active *Ganoderma lucidum* Compounds and Synthesis of Improved Derivatives That Confer Anti-cancer Activities *in vitro*

**DOI:** 10.3389/fphar.2019.00115

**Published:** 2019-02-19

**Authors:** Michelle M. Martínez-Montemayor, Taotao Ling, Ivette J. Suárez-Arroyo, Gabriela Ortiz-Soto, Camille L. Santiago-Negrón, Mercedes Y. Lacourt-Ventura, Anibal Valentín-Acevedo, Walter H. Lang, Fatima Rivas

**Affiliations:** ^1^Cancer Research Unit, Department of Biochemistry, School of Medicine, Universidad Central del Caribe, Bayamón, Puerto Rico; ^2^Department of Chemical Biology & Therapeutics, St. Jude Children’s Research Hospital, Memphis, TN, United States; ^3^Department of Biology, University of Puerto Rico, Bayamón, Puerto Rico; ^4^Department of Microbiology and Immunology, School of Medicine, Universidad Central del Caribe, Bayamón, Puerto Rico

**Keywords:** *Ganoderma lucidum*, ergosterol peroxide, breast cancer, EP derivatives, natural product

## Abstract

We previously reported that *Ganoderma lucidum* extract (GLE) demonstrate significant anti-cancer activity against triple negative inflammatory breast cancer models. Herein, we aimed to elucidate the bioactive compounds of GLE responsible for this anti-cancer activity. We performed NMR, X-ray crystallography and analog derivatization as well as anti-cancer activity studies to elucidate and test the compounds. We report the structures of the seven most abundant GLE compounds and their selective efficacy against triple negative (TNBC) and inflammatory breast cancers (IBC) and other human cancer cell types (solid and blood malignancies) to illustrate their potential as anti-cancer agents. Three of the seven compounds (ergosterol, 5,6-dehydroergosterol and ergosterol peroxide) exhibited significant *in vitro* anti-cancer activities, while we report for the first time the structure elucidation of 5,6-dehydroergosterol from *Ganoderma lucidum*. We also show for the first time in TNBC/IBC cells that ergosterol peroxide (EP) displays anti-proliferative effects through G1 phase cell cycle arrest, apoptosis induction via caspase 3/7 activation, and PARP cleavage. EP decreased migratory and invasive effects of cancer cells while inhibiting the expression of total AKT1, AKT2, BCL-XL, Cyclin D1 and c-Myc in the tested IBC cells. Our investigation also indicates that these compounds induce reactive oxygen species, compromising cell fate. Furthermore, we generated a superior derivative, ergosterol peroxide sulfonamide, with improved potency in IBC cells and ample therapeutic index (TI > 10) compared to normal cells. The combined studies indicate that EP from *Ganoderma lucidum* extract is a promising molecular scaffold for further exploration as an anti-cancer agent.

## Introduction

Increased toxic effects from conventional therapies, as well as evidence from recent reports that prove the efficacy of natural therapies, have caused a rise in their use by patients with cancer (Gao et al., 2003; Chen et al., 2006; Jin et al., 2016; Suárez-Arroyo et al., 2017). Among these therapies is the whole medicinal mushroom *Ganoderma lucidum*, which has been used in traditional Chinese medicine for more than two millennia (Paterson, 2006). Patients with cancer who take *Ganoderma lucidum* extract (GLE) display improved quality of life and prolonged lives without interference with their conventional therapy (Jin et al., 2016). The most common uses for commercially available GLE include prevention and treatment of hypertension, cancer, and immunological disorders (Liu et al., 2002). Although the fruiting body of *Ganoderma lucidum* has been used as a traditional medicine for decades, the spores have also become a research subject more recently (Min et al., 2000). The spores contain mainly lanostane-type triterpenes (Xie et al., 2006) and polysaccharides (Huie and Di, 2004) similar to those found in the fruiting body, which are the main chemical compounds to which anti-cancer activities of GLE are attributed.

Mechanisms of cancer prevention by GLE have been summarized in several reports (Jong and Donovick, 1989; Wasser and Weis, 1999). We have reported that commercially available whole mushroom GLE selectively inhibits breast cancer cell viability and in various models of human cancer induces apoptosis, reduces invasion, and regulates key signaling molecules (Martinez-Montemayor et al., 2011). Moreover, we have also shown that GLE reduces tumor volume in mice by ∼50% when administered alone (Suarez-Arroyo et al., 2013) or in combination with conventional therapy (Suarez-Arroyo et al., 2016) in mice xenografts. Thus, the aim of the present study was to elucidate the chemical constituents of GLE responsible for its biological activity and characterize their efficacy as single agents in various cancer cell models, particularly in inflammatory breast cancer. Herein we describe the structure elucidation of the 7 most abundant chemical components of GLE (whole mushroom ReishiMax) by NMR studies, X-ray crystallography and analog derivatization. Our work demonstrates the *in vitro* efficacy of these compounds, which include triterpenes, and sterols, in various cancer models. To overcome poor solubility properties, we synthesized improved derivatives, which display superior potency against aggressive models of breast cancer.

## Materials and Methods

### Experimental Chemistry Procedures

#### General Information

Capsules (500 mg) of commercially available whole mushroom ReishiMax GLp^TM^ (Pharmanex Inc., Provo, UT, United States), consisting of powdered *Ganoderma lucidum* extract (GLE) fruiting body and cracked spores were used (Martinez-Montemayor et al., 2011; Suarez-Arroyo et al., 2013, 2016). All manipulations were carried out under inert gas atmosphere unless otherwise noted. Anhydrous tetrahydrofuran (THF), diethyl ether (Et_2_O), dichloromethane (CH_2_Cl_2_), toluene (PhCH_3_), acetonitrile (CH_3_CN), methanol (MEOH), and dimethylformamide (DMF) were obtained from a solvent drying system. Reagents of the highest available quality were purchased commercially and used without further purification unless otherwise stated. Title compounds were purified by flash column chromatography using E. Merck silica gel (60, particle size 0.040–0.063 mmol) or Biotage Isolera Four with normal-phase silica gel. Reactions were monitored by thin-layer chromatography (TLC) on 0.25 mmol E. Merck silica gel plates (60F-254), using UV light for visualization and an ethanolic solution of anisaldehyde, or PMA, CAM solutions and heat as developing agents. Reactions were also monitored by using Agilent 1100 series LCMS and low-resonance electrospray ionization (ESI) model with UV detection at 254 nm. The structures of the synthesized compounds were confirmed by ^1^H and ^13^C-NMR that were recorded on 400/or 500 MHz Bruker AVANCE III HD NMR (see Supplementary Figures S9–S17). Chemical shifts were reported as ppm relative to the solvent residual peak (CHCl_3_: 7.26 ppm for ^1^H, 77.2 ppm for ^13^C; acetone-d_6_: 2.05 ppm for ^1^H, 29.9 ppm for ^13^C; Pyridine d_5_: 2.50 ppm for ^1^H, 39.5 ppm for ^13^C). Data are reported as follows: chemical shifts, multiplicity (s = singlet, d = doublet, t = triplet, q = quartet, quint = quintet, m = multiplet, br = broad), coupling constant (Hz), and integration. Data were processed by using MestReNova. Optical rotations were measured on a DCIF polarimeter (JASCO P-1010) using a 2-mL cell with a 100-mm path length. High-resolution mass spectra (HRMS) were recorded on an Agilent ESI-TOF (time of flight) mass spectrometer using matrix-assisted laser desorption ionization (MALDI) or electrospray ionization (ESI) or on a Waters Xevo G2 Q-ToF mass spectrometer. Compounds were analyzed by using ESI in positive-ion mode. The purity of each synthesized compound was determined on a Waters ACQUITY UPLC-PDA-ELSD-MS system using a C_18_ reverse phase column and 0.1% formic acid/water – 0.1% formic acid/acetonitrile as the solvents. All synthesized compounds were at least 95% pure based on analytical HPLC and NMR. Chemical yields refer to purified compounds (^1^H-NMR).

#### Bioactivity Fractionation of GLE (Fraction 1–100)

As previously described by Tu et al. (2010), preparative HPLC separations were performed on a Gemini 5-μm C18 110A column (30 mm × 50 mm, 5 μm, Phenomenex, Inc., Torrance, CA, United States). A Shimadzu LC-8A binary preparative pump with a Shimadzu SCL-10A VP system controller was connected to the Gilson 215 auto sampler and Gilson 215 fraction collector (Gilson, Inc., Middleton, WI, United States). Detections were performed by a Shimadzu SPD-M20A diode-array detector and a Shimadzu ELSD-LT II evaporative light-scattering detector (Shimadzu Corp., Kyoto, Japan). The mobile phase consisted of water (A) and Acetonitrile (B): 0 min, 98:2; 0.5 min, 98:2; 6.5 min, 0:100; 12.3 min, 0:100; 12.5 min, 98:2; 12.95 min, stop. The flow rate was 25 mL/min. Briefly, fractions (A – fractions 1–6, B – fractions 7–9, C – fractions 10–17, D – fractions 18–31, E – fractions 32–41, F – fractions 42–100) were collected and combined based on mass spectra data; their TLC profiles and biological properties were evaluated (Supplementary Figure S1).

#### Crystal Structure of Ergosterol (Compound 4) and 5,6-Dehydroergosterol (Compound 5)

Fraction 98 and 99 (out of 103 fractions collected) precipitated and crystals were collected for single crystal diffraction studies conducted on a Bruker Kappa APEX-II CCD diffractometer equipped with Cu K_α_ radiation (λ = 1.5478). Crystals of the subject compound were grown by dissolving approximately 1 mg of sample in 350 μL of Ethyl Acetate, which was then vapor diffused with Pentane over several days. A 0.114-mm × 0.085 -mm × 0.076-mm piece of a colorless block was mounted on a Cryoloop with Paratone oil. Data were collected in a nitrogen gas stream at 100(2) *K* using ϕ and ϖ scans. Crystal-to-detector distance was 40 mm using variable exposure times (10–60 s) depending on θ, with a scan width of 1.0°. Data collection was 96.2% complete to 67.614° in θ (0.83 Å). A total of 37758 reflections were collected covering the indices, -12 <= *h* <= 12, -8 <= *k* <= 8, -40 <= *l* <= 40. A total of 8970 reflections were found to be symmetry independent, with an *R*_int_ of 0.0582. Indexing and unit cell refinement indicated a primitive, monoclinic lattice. The space group was found to be *P*_21_. The data were integrated by using the Bruker SAINT software program and scaled by using the SADABS software program. Solution by direct methods (SHELXT) produced a complete phasing model consistent with the proposed structure.

All non-hydrogen atoms were refined anisotropically by full-matrix least-squares (SHELXL-2014). All hydrogen atoms were placed using a riding model. Their positions were constrained relative to their parent atom by using the appropriate HFIX command in SHELXL-2014. The absolute stereochemistry of the molecule was established by anomalous dispersion using the Parson’s method with a Flack parameter of 0.002(230). Crystallographic data have been deposited at the Cambridge Crystallographic Data Center (CCDC number 1442028) and additional information provided in Supplementary Tables S1–S6.

### Experimental Cellular Procedures

#### Cell Culture

Cell lines were purchased from American Type Culture Collection (ATCC) or Leibniz-Institute Deutsche Sammlung von Mikroorganismen und Zellkulturen GmbH (DSMZ) and cultured without antibiotics or as specified by the provider. All cell lines were incubated at 37°C and were maintained in an atmosphere containing 5% CO_2_ according to proper sterile cell culture practices (Hay et al., 1992). The patient-derived triple-negative IBC cell line SUM-149 (Ethier et al., 1996) and the ER/PR-negative/HER2-positive SUM-190 cell line (kind gifts from Dr. Steven Ethier, Medical University of South Carolina; both available at Asterand Bioscience, Detroit, MI, United States) were cultured in Ham’s F12 medium (Life Technologies, Carlsbad, CA, United States) with 10% fetal bovine serum (FBS) as in Martinez-Montemayor et al. (2011), Suarez-Arroyo et al. (2016) and in Ham’s F12 medium with 2% FBS, respectively. Adherent cells were grown to 80–90% confluence, unless otherwise specified, and suspension cells were grown to densities recommended by Asterand, ATCC, or DSMZ before use. BJ (CRL-2522, normal human foreskin fibroblast), MCF-7 (HTB-22, human breast carcinoma) were cultured in EMEM medium (Life Technologies, Carlsbad, CA, United States) with 10% FBS, and MDA-MB-231 (HTB-26, human breast carcinoma, triple-negative breast cancer) cells were cultured in DMEM medium (Life Technologies, Carlsbad, CA, United States) supplemented with 10% FBS. Suspension cells KOPN8 (infant human B cell precursor acute lymphoblastic leukemia with MLL-MLLt1/ENL fusion), UoC-B1 (kindly gifted by Dr. William Evans of St. Jude Children’s Research Hospital), SUP-B15 (ACC 389), NALM-06 (ACC 128) or BCR-ABL (murine B cell precursor acute lymphoblastic leukemia of pediatric relapsed ALL with BCR-ABL fusion) cells were cultured in RPMI media supplemented with 10% FBS. Cells were tested with MycoAlert^TM^ Mycoplasma detection kit (Lonza) by using the manufacturer’s conditions and were deemed negative. The cell lines were authenticated by IDEXX BioResearch (Columbia, MO, United States).

#### CellTiter-Glo^®^ Viability Assay (CTG)

Either 1 × 10^3^–4.8 × 10^3^ or 4 × 10^2^–1.2 × 10^3^ cells were seeded to each well of 96- or 384-well white polystyrene flat-bottomed plates (3610 or 8804BC, Corning) in 100 μL or 30 μL media per well, respectively. The concentrations used were experimentally determined to ensure logarithmic growth during the duration of the experiment and prevent adverse effects on cell growth by DMSO exposure. The plates were incubated at 37°C in 5% CO_2_ for 24 h before treatment. Stock solutions of test compounds (10 mM in DMSO) in nine 3-fold serial dilutions were dispensed via pintool (Biomek). The final concentration of DMSO was 0.3% (v/v) in each well. The plates were incubated for 72 h at 37°C in 5% CO_2_ and then quenched with CellTiter-Glo^®^ reagent (50 μL/well in 96-well plate, 30 μL/well in 384-well plate) at RT. The positive controls included staurosporine (10 μM), gambogic acid (10 μM), and a toxic quinoline generated in-house. Plates were then incubated at RT for 20 min and centrifuged at 1000 rpm for 1 min. The cytotoxicity assay was performed by using the CellTiter-Glo^®^ Luminescent Cell Viability Assay kit (G7570, Promega, Madison, WI, United States) according to the manufacturer’s instructions. Luminescence was recorded in an Envision plate reader (PerkinElmer).

#### ApoTox-Glo^TM^ Triplex Assay

Cells (9.5 × 10^4^/well in 75 μL of fresh medium) were dispensed in 96-well black flat bottom (8807BC, Corning) plates. The cells were incubated for 12 h at 37°C and then treated with compounds (25 μL) for 24 h. DMSO was used as a negative control, and staurosporine was used as the positive control. The experiment was stopped by adding the Viability/Cytotoxicity Reagent and briefly mixed by orbital shaking (300–500 rpm for ∼30 s). Plates were incubated for 30 min at 37°C, and fluorescence was measured in an Envision plate reader at the following two wavelength sets: 400Ex/505Em (Viability) 485Ex/520Em (Cytotoxicity). Then, the Caspase-Glo^®^ 3/7 Reagent was added; plates were mixed briefly by orbital shaking (300–500 rpm for ∼30 s), followed by an additional 30 min incubation at RT. Then luminescence, which correlates with caspase 3/7 activation, was recorded with a plate reader to detemine apoptosis induction.

#### Cell Viability Assays

Cell viability assays (MTT or PI staining assays) in SUM-149, SUM-190, MDA-MB-231, and MCF10A cells were performed as we previously described (Suarez-Arroyo et al., 2016).

#### Colony Formation Assay

SUM-149 cells (1.5 × 10^6^ cells/well) were plated on a 6-well plate. Two days later, cell media was changed to 5% FBS, and cells were incubated at 37°C in a 5% CO_2_ atmosphere for 1 h before vehicle (0.1% DMSO) or EP (5–40 μM) was added. After 72 h of treatment, cells were trypsinized and reseeded at 200 cells/mL per well in 24-well plates. After 10 days of culture, colonies were visualized by crystal violet staining, and the colony numbers were counted under a microscope.

#### *De novo* In-Cell Protein Synthesis

First, 2.5 × 10^4^ cells/well were seeded in 8-well μ-slides (Ibidi^®^, #80826) and incubated at 37°C for 12 h. The cells were treated with vehicle, compounds Ganoderic acid A or EP, or cycloheximide (positive control) for 2 h to determine protein synthesis as nascent proteins generated using Biovision’s EZClick^TM^ Global Protein Synthesis Assay Kit, (#K715-100, Milpitas, CA, United States). The assay includes a robust chemical method based on an alkyne containing *o*-propargyl-puromycin (OP-puro) probe, which stops translation by forming covalent conjugates with nascent polypeptide chains. Truncated polypeptides are rapidly turned over by the proteasome and can be detected based on the subsequent click reaction with a fluorescent azide. The reaction was conducted according to the manufacturer’s protocol. Cells were imaged at 20× with a Nikon C2 scanning confocal microscope, and the whole image montage was quantified by using Gen5 Software and Lionheart^TM^ FX Automated Microscope (BioTek, Winooski, VT, United States).

#### Reactive Oxygen Species (ROS) Measurement

SUM-149 or MDA-MB-231 were plated in 96-well plates at a density of 2 × 10^4^ and 1 × 10^4^ cells/well, respectively, in black 96-well clear bottom tissue culture plates and incubated for 24 h at 37°C. The mRuby-Mito-7 plasmid was obtained from Addgene (#55874 from Michael Davidson). A stable clone of MCF-7 was generated by using Fugene 6 (Roche) to transfect cells (2 × 10^5^) with plasmid (2.0 μg) under neomycin selection. A stable cell line was selected by using G418 (300 μg/mL) for 2 weeks, sorted by flow cytometry, and maintained under G418 thereafter. Transfected MCF-7 cells were plated at a density of 2 × 10^4^ cells/well in black 96-well clear bottom tissue culture plates and incubated overnight at 37°C. To test for ROS formation and remove background, cells were replenished with fresh PBS with 2% FBS and treated with the desired compounds for 1 h at 37°C. DMSO (0.5%) was used as a negative control, while Menadione (Mena, at 10 μM) or *t*-Butyl hydroperoxide (TBHP) at 100 μM final concentration were used as a positive control. To evaluate the compounds’ effects on ROS formation inhibition, the free radical scavenger *N*-acetyl cysteine (NAC) was used. Cells were treated with or without NAC (500 μM or 100 μM) for 1 h, then CellROX^®^ green reagent (Molecular Probes #C10444) was added to a final concentration of 5 μM, and cells were incubated for an additional 30 min at 37°C. Cells were then washed 2× with PBS, followed by fixation with 4% paraformaldehyde (v/v) for 15 min, and 2 more washes with PBS. Fluorescence intensity was measured in a Clariostar^®^ plate reader (BMG LABTECH, Cary, NC, United States) at 535 nm.

#### Cell Cycle Assay

SUM-149 cells were treated for 48 h with EP, then collected and washed in 1X PBS. The cells (5 × 10^5^) were fixed and permeabilized in 70% ethanol at -20°C until further analysis. To measure the DNA content, the cells were washed and resuspended in 150 μL of PBS buffer then incubated in 100 μL of 100 μg/mL RNase A (MilliporeSigma, Burlington, MA, United States) for 15 min at RT, then 250 μL of 50 μg/mL Propidium Iodide (MilliporeSigma) was added. Samples were incubated at RT for 10 min and analyzed on a BD FACS Canto II flow cytometer (BD Biosciences, San José, CA, United States). Data were analyzed by using FlowJo software (V10, FlowJo, LLC, Ashland, OR, United States).

#### Annexin V Apoptosis Detection

SUM-149 cells (5 × 10^5^) were seeded and treated either with vehicle (0.1% DMSO) or with 20 μM of EP or with puromycin (200 ng/mL) as positive control for 48 h. After treatment floating cells were collected, and all cells were harvested and counted. Treated cells were analyzed for cell death by using the FITC Annexin-V/7AAD Apoptosis detection kit (product #640922, Biolegend, San Diego, CA, United States) according to the manufacturer’s instructions. Briefly, cells were resuspended in Annexin Binding Buffer, stained with Annexin V-FITC and/or 7-AAD, and incubated for 30 min at 4°C. Data were acquired on a BD FACS Canto II cytometer (BD Biosciences, San José, CA, United States) and analyzed by using FlowJo software (V10, FlowJo, LLC, Ashland, OR, United States).

#### Migration and Invasion Assays

Cell migration and invasion were measured by using Corning^®^ FluoroBlok^TM^ Cell Culture Inserts and performing BD BioCoat Matrigel invasion assays (BD Biosciences, San José, CA, United States) as described in Martinez-Montemayor et al. (2011) and Suarez-Arroyo et al. (2016).

#### Immunoblots

Breast cancer cells treated with vehicle or EP were lysed, and equal total protein was resolved via SDS-PAGE and immunoblotted with indicated antibodies (Cell Signaling Technologies, Abcam, Sigma) as described previously (Castillo-Pichardo et al., 2009; Martinez-Montemayor et al., 2011; Suarez-Arroyo et al., 2016).

### Statistical Analysis

For the CTG assay, 3 or 4 replicate assays were conducted for each experimental condition, and a minimum of 3 independent experiments were conducted for cellular assays. The mean luminescence of each experimental treatment group was normalized as a percentage of the mean intensity of untreated controls. EC_50_ values (μM) were calculated by Pipeline Pilot Software (Accelrys, Enterprise Platform, San Diego, CA, United States). EC_50_ values (μM) from the viability assay were calculated from dose response curve-fitting via non-linear regression by using GraphPad Prism (Version 7.0, San Diego, CA, United States). For cell viability, colony formation, cell-cycle progression, cell death, and western blot assays, the analyses were performed via one-way or two-way ANOVA with post-testing for each condition (concentration, cell cycle stage, apoptotic event, cell line) by using GraphPad Prism.

## Results

### GLE Chemical Constituents

Most mushrooms contain about 90% water by weight, and the remaining 10% consists of protein, fat, carbohydrate, fiber, vitamins, and minerals (Borchers et al., 1999). Medicinal mushrooms such as *Ganoderma lucidum* also contain a variety of bioactive molecules, namely triterpenes, steroids, phenols, nucleotides, glycoproteins, and polysaccharides. Triterpenes, polysaccharides, and peptidoglycans are the 3 major physiologically active constituents in *Ganoderma lucidum* (Boh et al., 2007). Our initial screening revealed that only a few fractions induced selective cell death in cancer cell lines (see Supplementary Figure S1). However, additional unidentified compounds in the fractions at minute quantities might also possess anti-cancer activity or interact synergistically with the identified compounds. For those initial screenings, the total anti-cancer activity of each fraction (i.e., fraction A – F), rather than the anti-cancer activity of a single molecule was monitored. Further characterization and purification of the bioactive components yielded several abundant compounds from a total of 100 fractions (see Supplementary Information). GLE was extracted via Soxhlet extractor for 24 h in isopropanol (1 mL/10 mg). Bioassay-guided fractionation of the crude extract indicated the presence of tetracyclic triterpenes from ganoderic acid and ergosterol (compounds 1–6) series (by TLC and MS) and lipids (compound 7).

**FIGURE 1:**
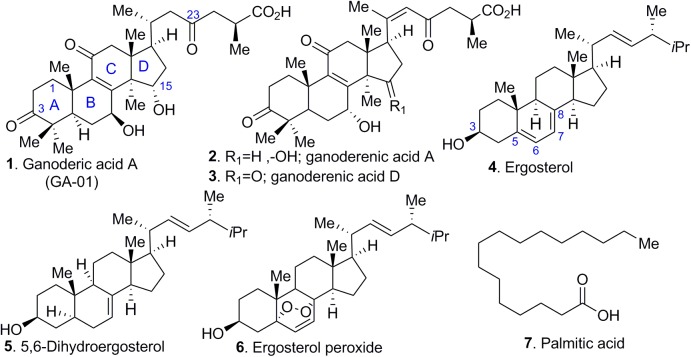
Selected chemical constituents of GLE. Compounds extracted from *Ganoderma lucidum* include (1) ganoderic acid A, (2, 3) ganoderenic acid A and D, (4) ergosterol, (5) 5, 6-dihydroergosterol, (6) ergosterol peroxide, and (7) palmitic acid.

Further characterization of compounds 1–7 (Figure 1) revealed that palmitic acid (compound 7) was the most abundant compound in the extract by weight. Although these compounds are reported in the literature, their purification poses challenges, particularly for the ganoderic acids, which share similar retention times. In fact, to obtain pure ganoderic acid A (GA-01, compound 1), we resorted to generating the methyl ester of ganoderic acid A (GA-01-ME) (see Figure 9, compound 1a), which could be easily separated from the unidentified compounds. Hydrolysis of the methyl ester with base provided GA-01 as an amorphous solid, in agreement with reported characterization data (see Supplementary Information). Ganoderenic acid A and Ganoderenic acid D (compounds 2–3, respectively) were isolated in minute quantities because they overlap with other isomers, co-eluted in the silica gel column, and did not yield enough material for subsequent biological studies. Repeated column chromatography provided 3 sterols; their spectroscopic properties (^1^H- and ^13^C-NMR) confirmed that they were ergosterol (compound 4), 5,6-dehydroergosterol (compound 5), and ergosterol peroxide (compound 6), see Figure 1.

**FIGURE 2:**
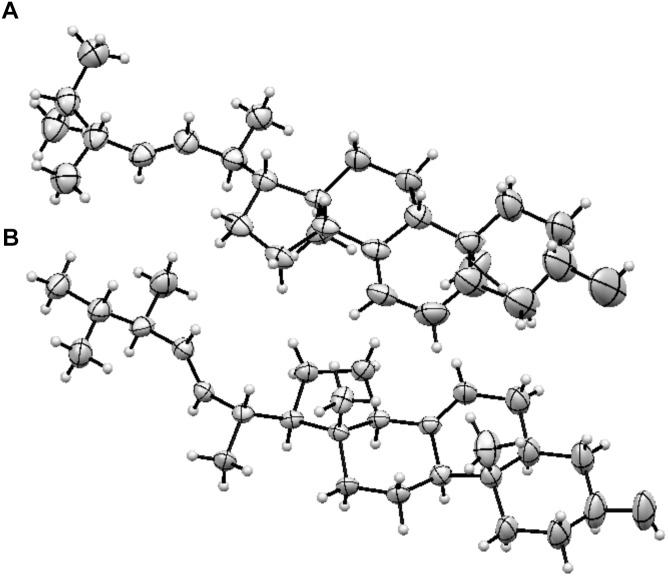
ORTEP structures **(A)** Ergosterol, **(B)** 5, 6-dihydroergosterol.

Ergosterol is the major fungal membrane sterol that regulates fluidity and plasma membrane biogenesis and function (Zhang and Rao, 2010). Importantly, the structures of ergosterol and 5,6-dehydroergosterol were further confirmed by subjecting them to X-ray crystallography. Although several X-ray crystal structures have been reported for ergosterol (Bernal and Crowfoot, 1935; Bernal et al., 1940; Hull and Woolfson, 1976; Cantrell et al., 1999; Guo et al., 2004), our studies report the X-ray structure of 5,6-dehydroergosterol, which has not been previously reported. The ORTEP structures of the dimeric unit cell are depicted in Figure 2; ball-and-stick representations of the X-ray molecular structures are shown in Supplementary Figure S8. Crystallographic data are summarized in detailed parameters in Supplementary Tables S1–S6.

The structure elucidation of ergosterol peroxide (EP, compound 6), the most biologically active compound, was confirmed by mass spectrometry coupled with 2D NMR experiments, namely COSY (^1^H-^1^H correlation), HMQC (^1^H-^13^C correlation), HMBC (^1^H-^13^C correlations), and NOESY, which identified the structure as ergosterol peroxide (EP). Both physical and spectroscopic data of EP are in full agreement with those previously reported for ergosterol peroxide (Nam et al., 2001).

### GLE Chemical Constituents Decrease Cancer Cell Viability

To investigate whether purified GLE compounds exert anti-cancer activities, we performed cell viability assays in breast cancer (MDA-MB-231 and MCF-7) and inflammatory breast cancer (IBC, SUM-149, and SUM-190) models. We also evaluated the compounds’ activity in human and murine leukemia (KOPN8, BCR-ABL, UoCB-1, SUP-B15, and NALM06) cell lines. As a control, normal human skin fibroblasts (BJ) cells and non-cancerous mammary epithelial cells (MCF10A) were used.

**FIGURE 3:**
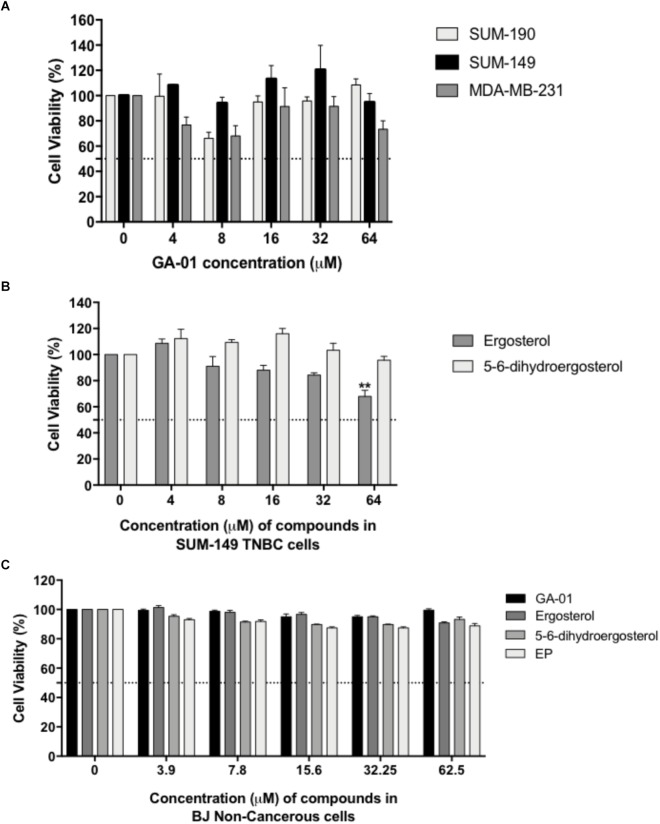
Effect of GA-01, ergosterol, 5, 6-dihydroergosterol, and ergosterol peroxide in breast cancer cell lines and normal fibroblasts. SUM-149, MDA-MB-231, SUM-190, and BJ cells were seeded and treated as described in the materials and methods section. **(A)** GA-01 had an EC_50_ > 50 μM in various breast cancer cell lines. **(B)** Ergosterol, and 5, 6-dihydroergosterol had an EC_50_ > 50 μM, and ergosterol significantly reduced SUM-149 cell viability at 64 μM. **(C)** BJ cell viability was not affected by the compounds at the concentrations tested. Bars represent mean ± SEM of at least three biological replicates. ^∗∗^*P* < 0.01 compared to vehicle.

We tested different concentrations of GLE-extracted bioactive chemical components for 72-h treatment periods. Dose-dependent studies of the identified GLE-extracted chemical components using CellTiter-Glo^®^ or PI staining viability assays showed modest activity of GA-01 and 5,6-dehydroergosterol. Their efficacy is at the higher micromolar range (>50 μM) for various breast cancer cell lines (Figure 3A) under the tested conditions. Ergosterol significantly reduced cancer cell viability starting at 64 μM in breast cancer cellular models (Figure 3B). Importantly, GA-01, 5,6-dehydroergosterol, ergosterol or EP did not induce cytotoxic effects in normal BJ cells (Figure 3C). Moreover, palmitic acid did not show activity at the tested concentrations in the evaluated cell lines (Supplementary Figure S2).

**FIGURE 4:**
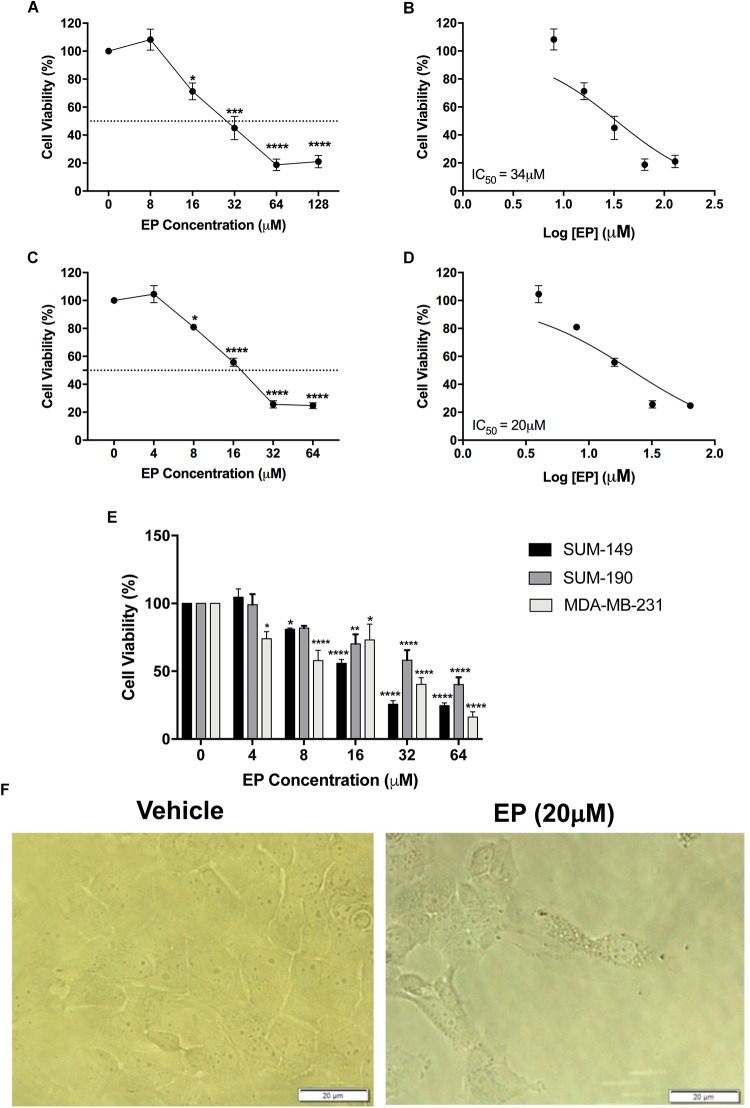
Effect of ergosterol peroxide (EP) in breast cancer cell lines. SUM-149, MDA-MB-231, and SUM-190 cells were seeded and treated as described in the Section “Materials and Methods.” **(A,B)** SUM-149 cells treated with EP for 24 h significantly decreased cell viability. **(C,D)** Treatment with EP for 72 h significantly reduced SUM-149 cell viability beginning at 8 μM, EC_50_ = 20 μM. **(E)** Comparison of SUM-149, MDA-MB-231, and SUM-190 cell viability after treatment with EP shows reduced viability in all breast cancer cells. **(F)** Cell morphology changes and detection of vacuoles in SUM-149 upon EP treatment. Bars represent mean ± SEM of at least 3 biological replicates. ^∗^*P* < 0.05, ^∗∗^*P* < 0.01, ^∗∗∗^*P* < 0.001, ^∗∗∗∗^*P* < 0.0001 compared to vehicle.

From the seven isolated bioactive compounds, EP showed the greatest anti-cancer activity. We show for the first time that EP induces a time- and concentration-dependent decrease (*P* < 0.05) in SUM-149 TNBC/IBC cell viability, with reported EC_50_ values of 34 and 20 μM at 24 and 72 h, respectively (Figure 4A–D). Moreover, our results for MDA-MB-231 TNBC cells and SUM-190 IBC cells also show inhibition of viability by EP in a concentration-dependent manner, with the EC_50_ being 19 and 43 μM, respectively (Figure 4E). These results suggest that TNBC cells might display higher sensitivity to EP. SUM-149 cell number was reduced, while morphology was affected, and the presence of vacuoles was detected upon EP treatment (Figure 4F). EP also displayed reduced viabilities, with reported EC_50_ values ranging from 7 to 22 μM in additional cancer cellular models (Supplementary Figure S2), whereas in normal human fibroblast BJ cells (Figure 3C) and MCF10A non-cancerous mammary epithelial cells (Supplementary Figure S2), EP did not induce cytotoxicity. Therefore, EP exerts selective effects on cancer cell viability, similar to the effects we obtained with whole mushroom extract (GLE), indicating an ample therapeutic window for this compound (Martinez-Montemayor et al., 2011; Dai et al., 2017).

### EP Induces Cell Cycle Arrest and Apoptosis

**FIGURE 5:**
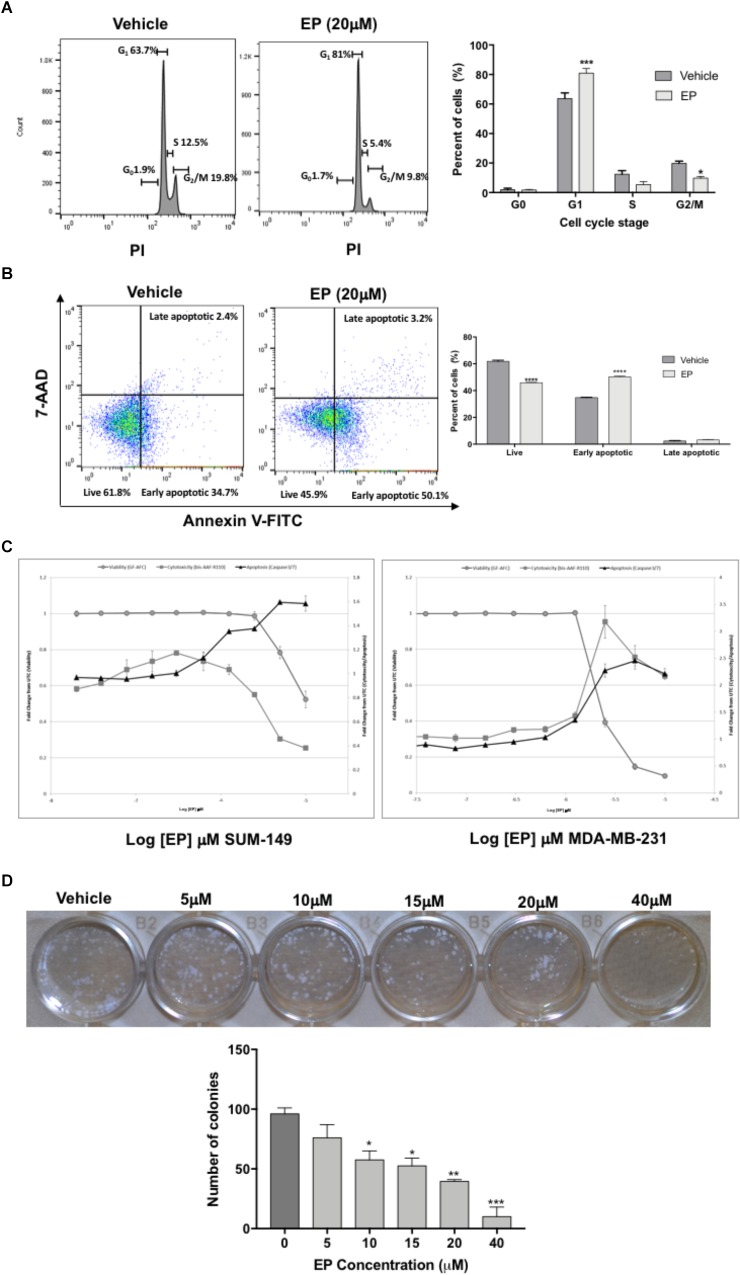
Effect of EP on SUM-149 breast cancer cells. **(A)** A cell-cycle progression assay of cells treated with EP for 48 h shows an increased percentage of cells in G1 and decreased percentage of cells in G2/M. **(B)** Cell death assay shows decreased live cells and an increased percentage of cells in early apoptosis after 48 h of EP treatment. **(C)** EP induces apoptosis via caspase 3/7 activity in breast cancer cells. The Triplex Glo assay was used to determine viable cells (GF-AFC, closed gray circles), compound cytotoxicity by membrane integrity (bis-AAF-RF110, closed gray squares) or apoptotic cells (caspase 3/7 activity, black triangles). Graphs show that EP decreases viability and increases apoptotic activity. **(D)** Colony formation assay in cells treated with EP for 72 h. Pictures were taken 10 days after pretreated cells were seeded at 200 cells/mL. Bars represent mean ± SEM. ^∗^*P* < 0.05, ^∗∗^*P* < 0.01, ^∗∗∗^*P* < 0.001, ^∗∗∗∗^*P* < 0.0001 compared to vehicle.

To determine whether the inhibition of cell viability induced by EP was due to effects on cell-cycle progression, we investigated the compound’s effects in SUM-149 TNBC/IBC cells. Our results show a significant effect of EP on cell-cycle stage in SUM-149 cells (Figure 5A). Specifically, we detected a significant (*P* < 0.001) increase in percentage of cells (∼20%) in G1 phase, with a significant reduction (*P* < 0.05) of the percentage of cells (∼10%) in G2/M, indicative of an arrest in G1.

Cells commonly fail to bypass the G1 phase of the cycle due to mutations or DNA damage, which can result in apoptosis. Hence, we sought to determine whether EP induces programmed cell death in SUM-149 cells. Treated cells were double-stained with Annexin V and 7-AAD dyes to determine the percentage of cells in early vs. late apoptosis as well as the percentage of viable cells. As shown in Figure 5B, the percentage of cells in early apoptosis (Annexin+, 7AAD-) significantly increased (*P* < 0.0001) and that of live cells decreased (∼15%; Annexin-, 7AAD-) after 48 h of EP treatment (*P* < 0.0001), confirming our expectation of apoptosis induction. In addition, we performed caspase 3/7 activity assays as an alternate method of apoptosis detection. Both GA-01 (Supplementary Figure S3) and EP (Figure 5C) increase caspase 3/7 activity and decrease viability of both SUM-149 and MDA-MB-231 TNBC cells, substantiating the death-inducing effects of GLE chemical bioactive constituents. Finally, we show a concentration-dependent decrease in the number and size of colonies formed upon EP treatment of SUM-149 cells (Figure 5D), further confirming the cell-cycle arrest and death-inducing effects of EP.

### EP Effects on Protein Synthesis and ROS Formation

**FIGURE 6:**
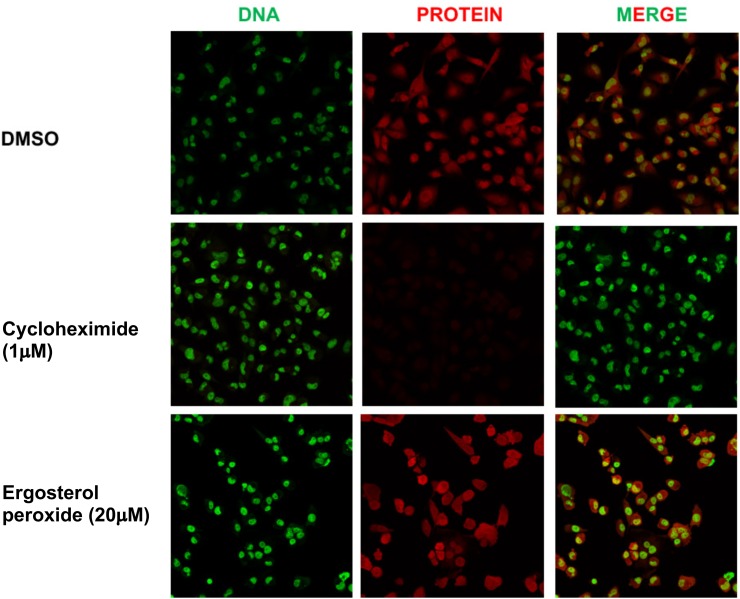
Click assay showing effects of EP on *de novo* protein synthesis in MDA-MB-231 breast cancer cells. (1) DMSO, (2) cycloheximide (1 μM), (3) EP (20 μM), 2 h. DNA stain (green). New protein (red). Merge (20×).

We previously showed that a 24 h treatment of whole mushroom GLE induces ∼50% protein synthesis inhibition in SUM-149 TNBC cells (Suarez-Arroyo et al., 2013; Suárez-Arroyo et al., 2017). To test whether GA-01 or EP derived from GLE induce *de novo* protein synthesis inhibition, we tested them in TNBC models following an in-cell-click *de novo* protein synthesis assay by treating cells for 2 h under the same conditions used for the known protein synthesis inhibitor cycloheximide (Liu et al., 2012). Our results show that EP and GA-01 do not inhibit *de novo* protein synthesis under these experimental conditions (Figure 6 and Supplementary Figure S4).

**FIGURE 7:**
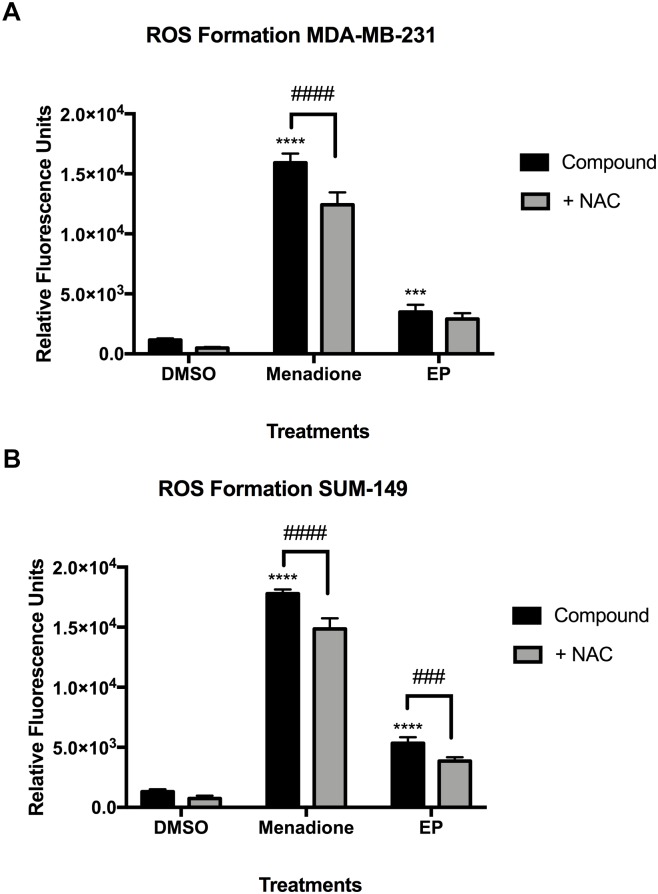
Reactive oxygen species (ROS) formation in MDA-MB-231 and SUM-149 cells. **(A)** MDA-MB-231 breast cancer cells or **(B)** SUM-149 breast cancer cells were treated as described in the Section “Materials and Methods” with EP (20 μM), or menadione (10 μM) as a positive control. *N*-Acetyl Cysteine (500 μM) was added to inhibit ROS formation. Bars depict mean ± SEM of triplicates. ^∗∗∗∗^*P* < 0.0001, ^∗∗∗^*P* < 0.001 compared to vehicle. ^####^*P* < 0.0001, ^###^*P* < 0.001 compared to NAC.

We next sought to determine whether the individual compounds affected reactive oxygen species (ROS) formation in SUM-149, MDA-MB-231 and in MCF-7 breast cancer cells. Live-cell CellROX^®^ assays for oxidative stress detection were conducted to evaluate whether GA-01, ergosterol, 5,6-dehydroergosterol and EP trigger intrinsic apoptosis pathways by inducing ROS. CellRox^®^ green reagent is a permeable dye with weak fluorescence while in a reduced state and exhibits bright green photostable fluorescence upon oxidation by ROS (Ling et al., 2018). We used menadione or *t*-Butyl hydroperoxide (TBHP) as positive controls (Figure 7 and Supplementary Figure S5) and co-treated with *N*-acetyl cysteine (NAC) to confirm that the signal was due to ROS formation induced by the compound. In MDA-MB-231 and SUM-149 cells, menadione treatment caused similar effects, where there was a significant reduction (*P* < 0.0001) in ROS formation in the presence of NAC of about 20%, which might indicate increased glutathione levels, directly interacting with ROS to generate a cysteine disulfide molecule or direct interference with the compound. EP significantly induced a twofold ROS formation (*P* < 0.001) for MDA-MB-231 (Figure 7A) and a fourfold ROS formation (*P* < 0.0001) in SUM-149 cells (Figure 7B). Importantly, there was a significant reduction (*P* < 0.001) in ROS formation of about 20% in SUM-149 cells in the presence of NAC, suggesting that EP’s effect is more pronounced in SUM-149 cells. In MCF-7 breast cancer cells, TBHP treatment reduced ROS formation in the presence of NAC by almost 50% (Supplementary Figure S5). GA-01, ergosterol, 5,6-dehydroergosterol and EP induced at least a twofold ROS increase; but unlike TBHP, only 9–25% ROS formation reduction was observed when co-treated with NAC, and none of these results were statistically significant (Supplementary Figure S5). Furthermore, we assessed the possibility of the endoperoxide motif to react with NAC, thus EP was incubated with NAC in DMSO for 24 h at 37°C. The reaction mixture was monitored by NMR and LC-MS and no observable olefin migration or opening of the endoperoxide was detected. Thus, the ability of EP to induce ROS could potentially be via a different mechanism from that of TBHP in MCF-7 cells.

### EP Reduces Cell Migration, Invasion, and Key Signaling Pathways in Breast Cancer Cells

**FIGURE 8:**
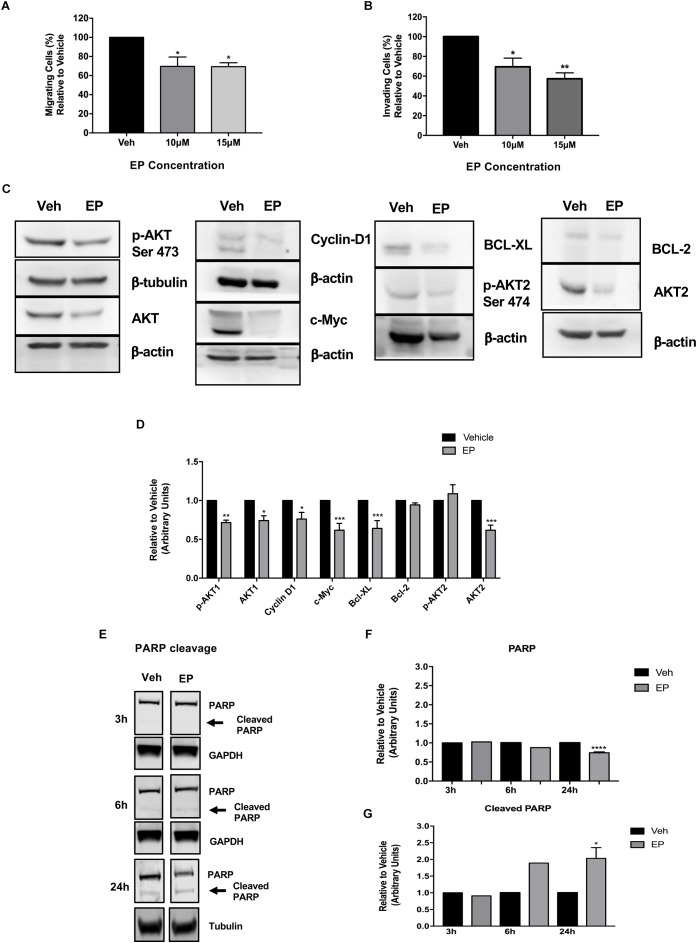
Ergosterol peroxide affects breast cancer cell migration and invasion and modulates cancer cell signaling. SUM-149 cells were treated with EP at various concentrations for 72 h as described in the materials and methods section. **(A)** EP decreases cancer cell migration. **(B)** There is a concentration-dependent decrease in cancer cell invasion. **(C)** EP modulates cancer cell survival, cell death, and proliferative signaling pathways in breast cancer cells. β-actin or β-tubulin was used as a loading control. **(D)** Densitometric analyses were done using Image J software. **(E)** EP (30 μM) induces PARP cleavage at 24 h of EP treatment. **(F)** Densitometric analysis of total PARP using Image J software. **(G)** Densitometric analysis of cleaved PARP using Image J software. All Western blots presented were cropped to improve clarity. Full-length blots are displayed in Supplementary Figure S7. Bars represent mean ± SEM of triplicates. ^∗∗∗∗^*P* < 0.0001, ^∗∗∗^*P* < 0.001, ^∗∗^*P* < 0.01, ^∗^*P* < 0.05 compared to vehicle. ^∗^*P* = 0.03 in cleaved PARP at 24 h compared to vehicle.

Previously, we have reported the anti-migratory activities of whole mushroom GLE in SUM-149 cells (Suarez-Arroyo et al., 2013, 2016; Suárez-Arroyo et al., 2017). To investigate EP’s effects on cell migration, we performed Boyden chamber assays; while to investigate invasion, Transwell chambers were used. Our results show that EP decreases cancer cell migration at lower doses (10 μM) than the reported EC_50_ (Figure 8A). EP shows a concentration-dependent decrease in invasion of cancer cells, as a significant reduction (*P* < 0.05) in invading cells was observed upon 10 μM treatment. Furthermore, an additional decrease (43%) was seen when cells were treated with a non-lethal dose of 15 μM of EP (*P* < 0.01) (Figure 8B). These results are the first to demonstrate the anti-migratory effects of EP in SUM-149 IBC cells.

Studies that focus on drivers of cancer cell survival and invasion signaling show the important role of the AKT isoforms in breast cancer. AKT1 and AKT3 are associated with breast cancer invasion (Lehman et al., 2012), and PTEN-deficient tumors depend on AKT2 for maintenance and survival (Chin et al., 2014). Our results show that EP treatment significantly decreases the expression of p-AKT1 (Ser 473, *P* < 0.01) and decreases total AKT1 (*P* < 0.05) and AKT2 levels (*P* < 0.001) (Figure 8C,D), while not affecting AKT3 levels in SUM-149 cells (Supplementary Figure S6). Regulation of AKT isoform expression by EP is extremely similar to what we reported with whole mushroom GLE in the same breast cancer cell lines (Suarez-Arroyo et al., 2016).

Because EP induced apoptosis, we investigated its effects on the expression on anti-apoptosis proteins BCL-2 and BCL-XL, which block the release of cytochrome C from the mitochondria. As shown in Figure 8C,D, EP significantly reduces the expression of BCL-XL (*P* < 0.001), as well as that of Cyclin D1 (*P* < 0.05) and c-Myc (*P* < 0.001), which are signaling molecules involved in cell cycle progression and proliferation in breast cancer cells. We further investigated the apoptosis inducing effects of EP in SUM-149 cells by monitoring PARP cleavage. Our results demonstrate EP significantly reduces total PARP levels (*P* < 0.0001) and induces PARP cleavage (*P* < 0.02) after 24 h of treatment in SUM-149 cells. Our combined results demonstrate the inhibitory effects of EP against cancer cells, affecting migratory and death-inducing processes, as well as the expression of key proteins that play vital roles in cancer progression.

### EP Derivatives

**FIGURE 9:**
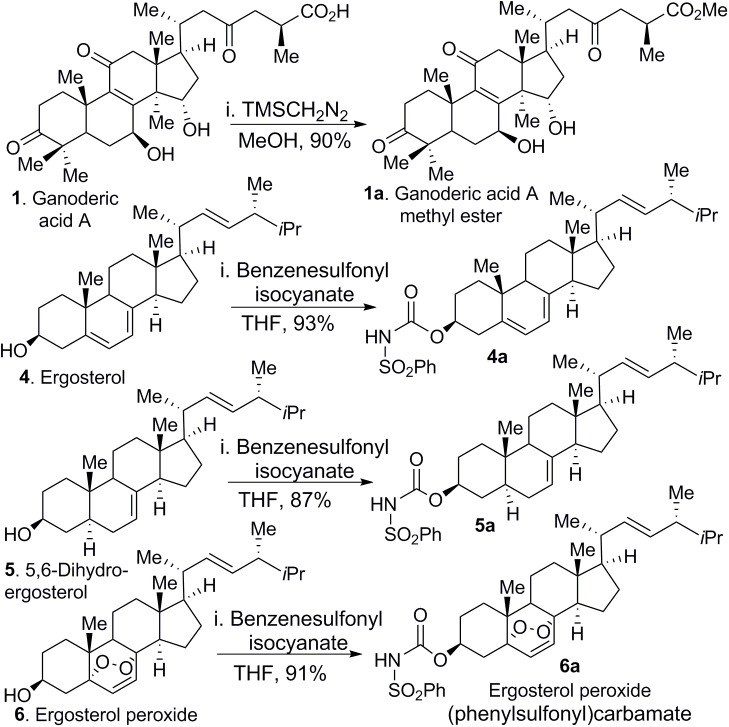
Chemical transformations of GA-01, ergosterol, 5, 6-dihydroergosterol and ergosterol peroxide to GA-01-ME (compound 1a), ergosterol sulfonamide (compound 4a), 5, 6-dihydroergosterol sulfonamide (compound 5a), and ergosterol peroxide sulfonamide (compound 6a).

To investigate whether chemical modification would improve biological activity of ergosterol, 5,6-dehydroergosterol and EP, we designed and synthesized their corresponding neutral C-3 benzenesulfonyl carbamate derivatives. We reasoned that the observed modest bioactivity of these compounds was partially due to compound solubility or membrane-permeability. Therefore, introduction of a carbamate functional group as a prodrug strategy should improve cellular activity. The synthesis of these derivatives is shown (Figure 9), which included the addition of benzenesulfonyl chloride (1.1 equiv.) to ergosterol (compound 4a, ergosterol sulfonamide), 5,6-dehydroergosterol (compound 5a, 5,6-dehydroergosterol sulfonamide), and EP (compound 6a, ergosterol peroxide sulfonamide) in THF at RT. The reaction was completed in good to excellent yields (87–93%). Weakly basic compounds can exist in the un-ionized or ionized form, depending on the pH of their environment and the pKa of the functional groups (-OH, -CO_2_H). It is frequently understood that ionized compounds have reduced permeability across lipid bilayers relative to that of un-ionized compounds. The neutral carbamate derivatives ergosterol sulfonamide, 5,6-dehydroergosterol sulfonamide, and EP sulfonamide should have improved solubility and cross the cell membrane, resulting in a favorable, slow intracellular accumulation.

**FIGURE 10:**
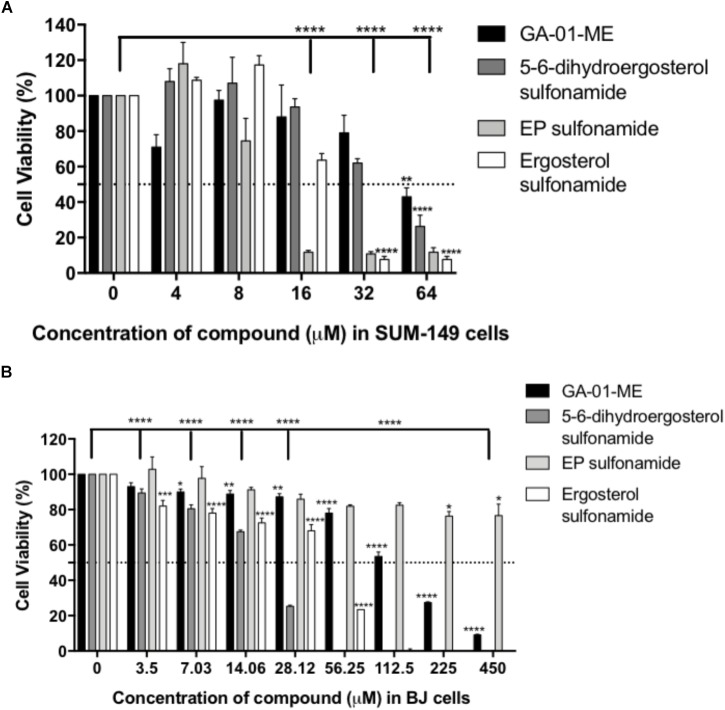
Effect of derivatives GA-01-ME, ergosterol sulfonamide, 5, 6-dihydroergosterol sulfonamide and ergosterol peroxide sulfonamide in **(A)** SUM-149 breast cancer cell viability and **(B)** normal BJ fibroblasts viability. Cells were seeded and treated as described in the Section “Materials and Methods.” Bars represent mean ± SEM of at least 3 biological replicates. ^∗∗∗∗^*P* < 0.0001, ^∗∗∗^*P* < 0.001, ^∗∗^*P* < 0.01, ^∗^*P* < 0.05 compared to vehicle.

The cytotoxicity evaluation of the compounds in breast cancer cells showed improved cytotoxicity effects over those of their corresponding parental structure (Figure 10A). EP sulfonamide shows a significant reduction in cancer cell viability (*P* < 0.0001) with an EC_50_ = 12 μM. Importantly, toxicity to human normal BJ fibroblasts was minimal when these cells were treated with EP sulfonamide, indicating that this compound selectively targets cancer cells (Figure 10B). In general, the rationale behind the use of prodrugs is to optimize the physiochemical properties of the compound while maintaining the same biological profile of the parental compounds. Further studies will be required to examine any differential effects across cell lines in a time-dependent manner and determine the octanol/water partition coefficient as a function of pH for these compounds.

## Discussion

Natural products from different sources such as fungi or plants continue to provide hit compounds with anti-cancer potential. Secondary metabolites from GLE include triterpenoids belonging to the ganoderic acid family, sterols (i.e., lanosterol, ergosterol, and ergosterol peroxide), lipids, flavonoids, and lignans. The ganoderic acids and the sterols share a similar molecular scaffold with the latter compounds displaying sparse higher oxidation states. Previous studies of these naturally occurring triterpenoids demonstrate that these compounds display a broad range of biological activity for multiple diseases, including cancer (Adamec et al., 2009; Thyagarajan et al., 2010; Thyagarajan-Sahu et al., 2011). Our previous studies evaluated the anti-cancer effect of the whole medicinal mushroom GLE (ReishiMax) that was used in the current study as starting material to purify, characterize, and investigate bioactive compounds (Martinez-Montemayor et al., 2011; Suarez-Arroyo et al., 2013, 2016; Suárez-Arroyo et al., 2017). The numerous compounds in this mushroom, including polysaccharides (Borchers et al., 1999; Wang et al., 2002; Zhang et al., 2002; Gao et al., 2003, 2005; Lin et al., 2003; Sliva, 2004; Lin, 2005; Paterson, 2006; Zhu et al., 2007; Joseph et al., 2011; Thyagarajan-Sahu et al., 2011) and triterpenes (Lee et al., 1998; Lin et al., 2003; Sliva, 2004; Jiang et al., 2008; Adamec et al., 2009; Thyagarajan et al., 2010; Thyagarajan-Sahu et al., 2011; Smina et al., 2011), are mainly responsible for its anti-cancer activities.

In the current study, the triterpene content in GLE ranged from 1 to 3%, but the lipid content remained consistent in three different batches. Triterpene content variations can occur due to differences in terpene production by mushroom or extraction methods. Herein, we present the seven most abundant compounds in GLE, which included ganoderic acid (GA-01), ganoderenic acid A, ganoderenic acid D, ergosterol, 5,6-dehydroergosterol, ergosterol peroxide (EP), and palmitic acid. Although previous crystal structures of ergosterol have been reported, in our current study we provide for the first time the X-ray structure of 5,6-dehydroergosterol from *Ganoderma lucidum*.

To capture potential differences in the pathobiology of cancer, we tested the effects of the isolated compounds in a representative panel of breast cancer cellular models. MDA-MB-231 and SUM-149 are triple-negative breast cancer (TNBC) cells that is, they lack the estrogen receptor (ER), progesterone receptor (PR), and human epidermal growth factor receptor 2 (HER2) and represent two distinct breast cancer subtypes: mesenchymal-like breast cancer (Yao et al., 2011) and epithelial-like inflammatory breast cancer (Zhang et al., 2009), respectively. SUM-190 is an IBC cell line that is ER/PR-negative but expresses the HER2 oncogene (Robertson et al., 2012); and MCF-7 cells express ER/PR receptors (ER/PR-positive/HER2-negative) (Brandes and Hermonat, 1983; Siddiqa et al., 2008). We focused our investigation on testing the compounds against the most aggressive breast cancer models (TNBC and IBC) because there is a lack of therapeutic agents for successful treatment in this patient cohort (Cristofanilli et al., 2007; Silvera et al., 2009; Fernandez et al., 2013).

Out of the seven isolated compounds, we show that ergosterol displayed modest biological activity against SUM-149 cells in agreement with previous ergosterol studies in HEPG2, MCF-7 and MDA-MB-231 cell lines (Bu et al., 2017). In the current study, EP displayed the greatest selective anti-cancer activity in the cell lines tested. EP features a cyclic peroxide (-O-O-), and these compounds are collectively known as endoperoxides. A classic example of this class of compounds is featured in the antimalarial drug, artemisinin, which has aid in reducing the widespread of drug-resistant parasites and has demonstrated cytotoxic activity (Posner et al., 2004). EP has been shown to have anti-cancer cell growth properties via regulation of various signaling pathways (e.g., AKT and c-myc) in a hepatocellular carcinoma cell model (Li et al., 2015, 2016). Similarly, our results show that EP selectively affects cancer cell viability in various cancer models, where SUM-149 IBC cells display the highest sensitivity. Interestingly, EP treatment in normal BJ and non-cancerous mammary epithelial MCF10A cells did not show significant cytotoxicity. Our studies revealed that EP induces G1 arrest in the cell cycle, which is accompanied with a decrease in cyclin D1 and c-myc expression and an increase in early apoptosis of SUM-149 cells. Comparable regulatory results were obtained in colorectal and hepatocellular cancer cell models upon EP treatment, with most cells stalled in the early stages of the cycle (Kang et al., 2015). Moreover, we show that EP reduces the expression of pro-survival p-AKT1, as well as levels of total AKT1 and AKT2 without affecting p-AKT2. It is possible that EP decreases total AKT2 without affecting p-AKT2 via a feedback mechanism, which induces signals to overcome the inhibitory effects obtained on total protein. Although p-AKT2 is not significantly upregulated, the phosphorylated levels might be stabilized to compensate for loss of total AKT2. Further studies are underway to understand the cell signaling regulatory effects of EP.

Interestingly, EP treatment of SUM-149 cells reduced cell viability and induced apoptosis detected by an increase in caspase-3/7 activity and PARP cleavage after 24 h of treatment. EP’s cytotoxicity profile in SUM-149 (TNBC/IBC) cells is consistent with that of cells exposed to a compound that induces cell-cycle arrest and early-phase apoptosis (Hook and Schagat, 2012). In contrast, we show that EP decreased cancer cell viability, while induced apoptosis via caspase 3/7 activation, and increased cytotoxicity of MDA-MB-231 TNBC cells. The combined results suggest the death-inducing effects of EP on the two TNBC cell lines occurs by different mechanisms, which we are currently studying. All the tested compounds induce ROS in breast cancer cells albeit at different levels. Specifically, EP increases ROS formation with greater capacity in SUM-149 cells, and the effects where reduced when co-treated with NAC. Therefore, intracellular ROS might be an additional contributing factor for the observed apoptotic effects of EP. Importantly, our results are consistent with the apoptosis-inducing effects of EP that have been previously obtained from distinct sources and demonstrated in various *in vitro* cancer models, including colorectal, prostate, and leukemia cells (Takei et al., 2005; Kobori et al., 2007; Russo et al., 2010; Kang et al., 2015).

Additional cancer cell properties that increase their pathogenicity include their ability to migrate and invade to distant organ sites. Thus, we investigated the regulatory effects of non-lethal doses of EP on SUM-149 cell motility and invasion. We show that EP displayed a reduction in cell migration and invasion, and these results are consistent with our previous studies with GLE wherein we demonstrated reduced invasion potential of breast cancer cells (Martinez-Montemayor et al., 2011; Suarez-Arroyo et al., 2013, 2016; Suárez-Arroyo et al., 2017). Moreover, our results presented in this study are the first to demonstrate the anti-migratory effects of EP in SUM-149 breast cancer cells, and we demonstrate anti-invasion effects at lower dosages than those reported in the literature in breast cancer lines (Li et al., 2016; He et al., 2018).

One of the liabilities with EP is sharing its core with cholesterol, an amphipathic molecule with part-water soluble, part-water insoluble character. Thus, while able to readily cross the cell membrane, the ability to deliver the pure compound faces challenges. EP’s solubility properties are poor in DMSO or water unless warmed up to 30°C. However, in this study, we showed that EP’s derivative, EP sulfonamide, was soluble at 10 mM in DMSO. Thus, we successfully demonstrate the generation of a selective anti-cancer compound with greater potency than EP, and ample therapeutic window when compared to BJ normal cells.

## Conclusion

Our study details the structure elucidation of the most abundant chemical constituents of whole mushroom *Ganoderma lucidum* and their efficacy in cancer models, particularly in triple negative inflammatory breast cancer. The isolated compounds of GLE, display biological activity across multiple cancer cell lines, with several degrees of potency. This investigation demonstrates for the first time (i) that EP as a single component of GLE, is the most biologically active compound against aggressive inflammatory breast cancer models with promising EC_50_ values in the low micromolar range and ample therapeutic index in normal cells. (ii) A detailed report of the X-ray crystal of 5,6-dehydroergosterol from *Ganoderma lucidum*; and (iii) that we developed improved derivatives of GA-01, ergosterol, 5,6-dehydroergosterol and EP by addressing their solubility. We also show that unlike many natural products, GA-01 and EP, do not act on proliferation by inhibiting protein synthesis. However, we show that cell death is mediated by caspase activation through the modulation of cancer dependent mechanisms (e.g., AKT pro-survival pathway). Our data indicates that GA-01, ergosterol, 5,6-dehydroergosterol and EP induced ROS in breast cancer cell models, suggesting that ROS mediated-mechanisms contribute in the onset of cell death signaling pathways. To better understand the cascade of signaling events we also demonstrate that EP modulates AKT, with subsequent reduction of proteins involved in cancer cell survival, proliferation, and progression (e.g., Cyclin D1, c-Myc). Finally, we have developed more potent compounds, such as EP sulfonamide, for further molecular mechanistic studies to understand the mode of action of these compounds in preclinical models.

## Author Contributions

MM-M and FR designed and performed the research, collected data, analyzed and interpreted data, performed statistical analysis, and wrote the manuscript. TL conducted GLE extraction, purification, structure elucidation, and derivatization. IS-A, WL, GO-S, CS-N, ML-V, and AV-A provided additional technical support to perform the experimental procedures. All authors provided input for the completion of the manuscript.

## Conflict of Interest Statement

The authors declare that the research was conducted in the absence of any commercial or financial relationships that could be construed as a potential conflict of interest.
