# Supplementary material for: Identification of Biologically Active Ganoderma lucidum Compounds and Synthesis of Improved Derivatives That Confer Anti-cancer Activities in vitro
Source: Front Pharmacol. 2019 Feb 19;10:115. doi: 10.3389/fphar.2019.00115 (PMC6389703; doi:10.3389/fphar.2019.00115)
Supplement: Supplementary file 1 [file Data_Sheet_1.docx]

**Supplementary Information**

Identification of Biologically Active *Ganoderma lucidum* Compounds and Synthesis of Improved Derivatives that Confer Anti-cancer Activities *in vitro*

Michelle M. Martínez-Montemayor^1*†^, Taotao Ling^2^, Ivette J. Suárez-Arroyo^1^, Gabriela Ortiz-Soto^1^, Camille L. Santiago-Negrón^3^, Mercedes Y. Lacourt-Ventura^1^, Anibal Valentín-Acevedo^4^, Walter H. Lang^2^, and Fatima Rivas^2*†^

^1^ Cancer Research Unit, Department of Biochemistry, School of Medicine, Universidad Central del Caribe, Bayamón, Puerto Rico, ^2^Department of Chemical Biology and Therapeutics, St. Jude Children’s Research Hospital, Memphis, TN, United States, ^3^Department of Biology, University of Puerto Rico, Bayamón, Puerto Rico, ^4^Department of Microbiology and Immunology, School of Medicine, Universidad Central del Caribe, Bayamón, Puerto Rico

^*^Email: [michelle.martinez@uccaribe.edu](mailto:michelle.martinez@uccaribe.edu)

Or [fatima.rivas@stjude.org](mailto:fatima.rivas@stjude.org)

**I) Additional experimental procedures** page

a) Supporting efficacy graphs (bio-guided extract), figures and blots 2-23

b) X-ray data for crystal structures of ergosterol (4) and 5,6-dehydroergosterol (5) 24-39

c) General experimental chemistry for compounds **1**-**7** 40-46

d) _1_H NMR and _13_C NMR NMR spectra data 47-55

**Data availability**

All data generated or analyzed during this study are included in this published article and its Supplementary Information files.

a) Supporting efficacy graphs (bio-guided extract), figures and blots **
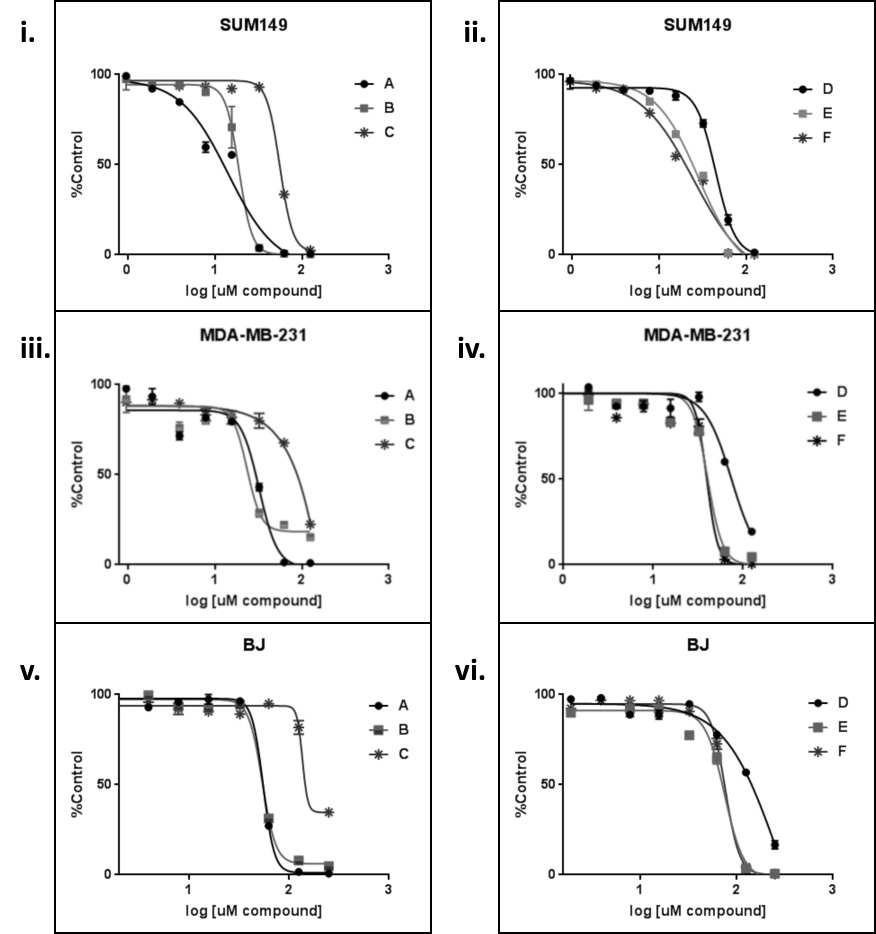
**

**Figure S1.** Evaluation of pooled fractions extracted from *Ganoderma lucidum* by Cell Titer Glo viability assay, 72-h treatment. Biological efficacy was tested in SUM-149 and MDA-MB-231 (breast cancer) and BJ (fibroblasts) cells. SUM-149 IBC cells are more sensitive than MDA-MB-231, but non-transformed BJ cells display higher EC_50_ values than do SUM-149 cells. Pooled fractions A= fraction 1-6, B= fraction 7-9, C= fraction 10-17), D= fraction 18-31, E= fraction 32-41, F= fraction 42-100. **i.** (EC_50_= 14.13 μM, 18.83 μM, and 55.62 μM respectively), **ii.** (EC_50_= 44.87 μM, 28.51 μM, and 25.14 μM, respectively), **iii,** (EC_50_= 32.2 μM, 23.36 μM, >50 μM, respectively), **iv,** (EC_50_= 74.27 μM, 40.42 μM, and 39.47 μM, respectively), **v.** (EC_50_= 54.99 μM, 53.86 μM, and 136 μM, respectively), **vi.** (EC_50_= 264 μM, 75 μM, and 76 μM, respectively).

**
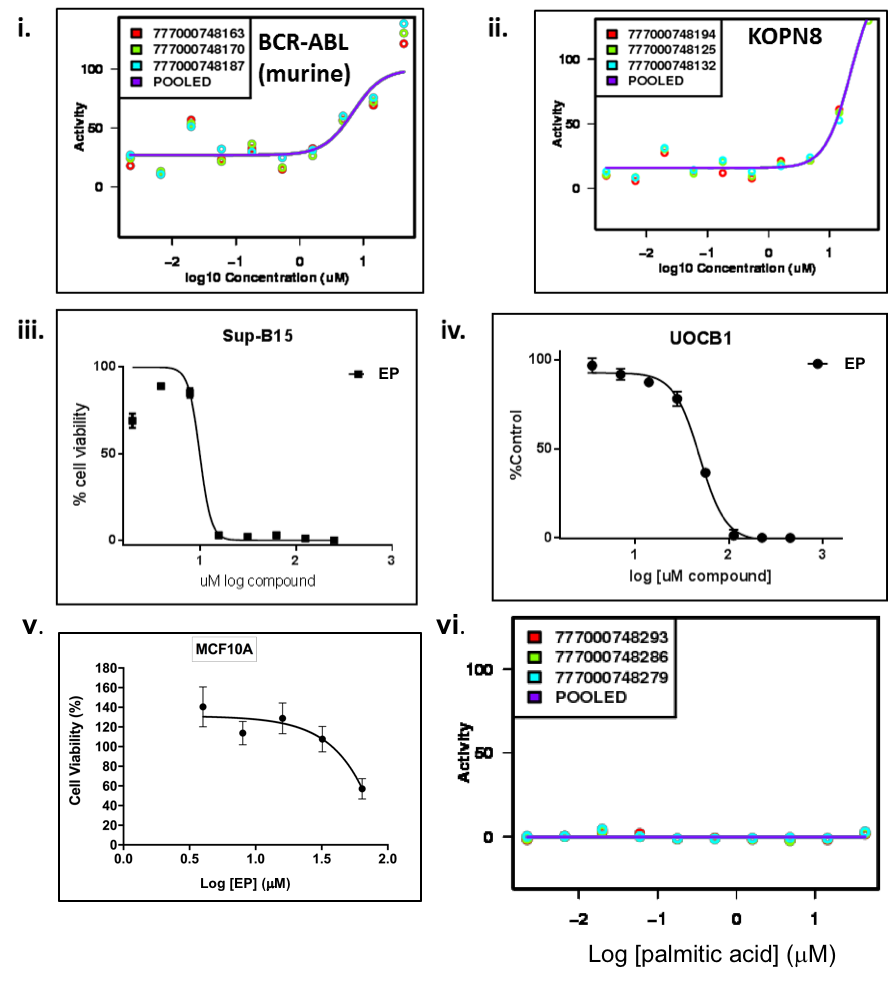
**

**Figure S2.** Evaluation of EP and palmitic acid by Cell Titer Glo viability assay (**i – iv.** and **vi.**) or PI staining (**v.**), 72-h treatment. **i.** BCR-ABL (EC_50_ =6.9 μM), **ii.** KOPN-8 (EC_50_ = 22.37 μM), **iii.** Sup-B15 (EC_50_ =9.86 μM), **iv.** Uocb-1 (EC_50_ =13.55 μM), **v.** MCF10A (EC_50_ > 100 μM), **vi.** Palmitic acid in BJ (EC_50_ > 100 μM). No detected biological activity was observed in the other cell lines at the tested concentration (≤100μM).


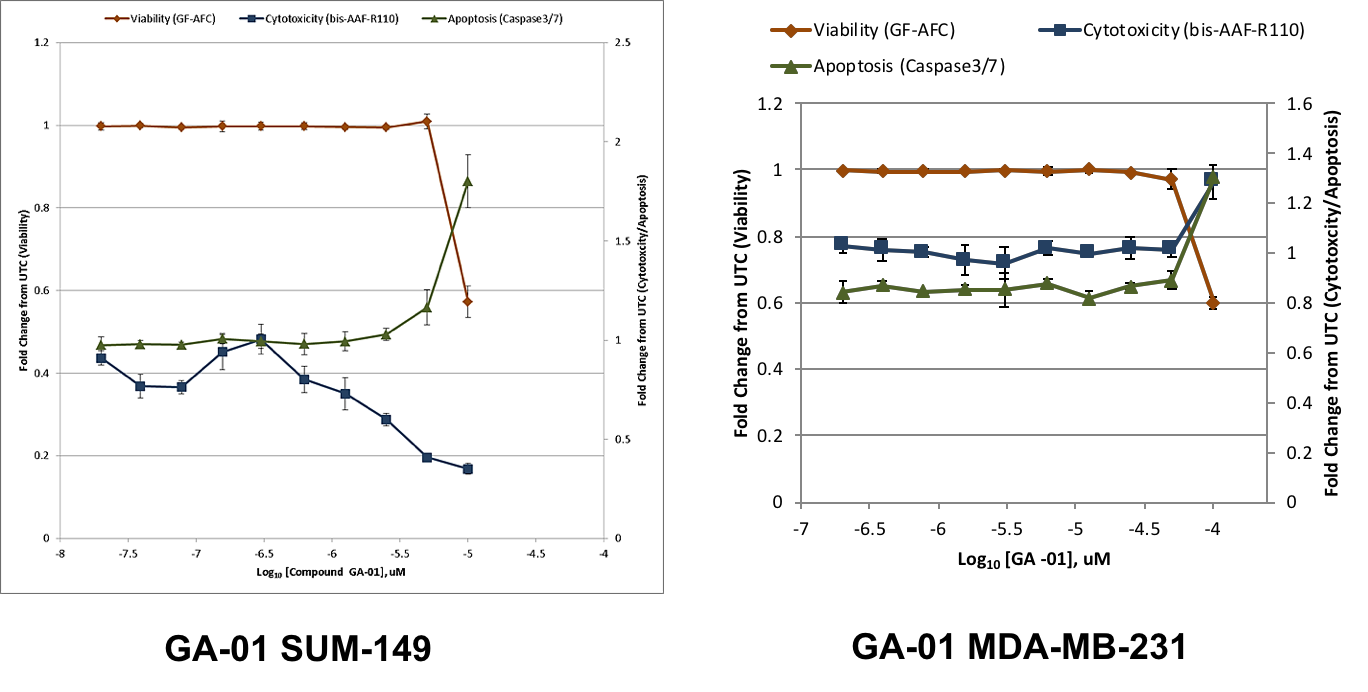


**Figure S3.** Effects of GA-01 on breast cancer cell apoptosis. The Triplex Glo assay was used to determine viable cells (GF-AFC), apoptotic cells (caspase 3/7), or compound cytotoxicity by membrane integrity (bis-AAF-RF110). Graphs show that GA-01 decreased viability while increasing apoptotic activity.


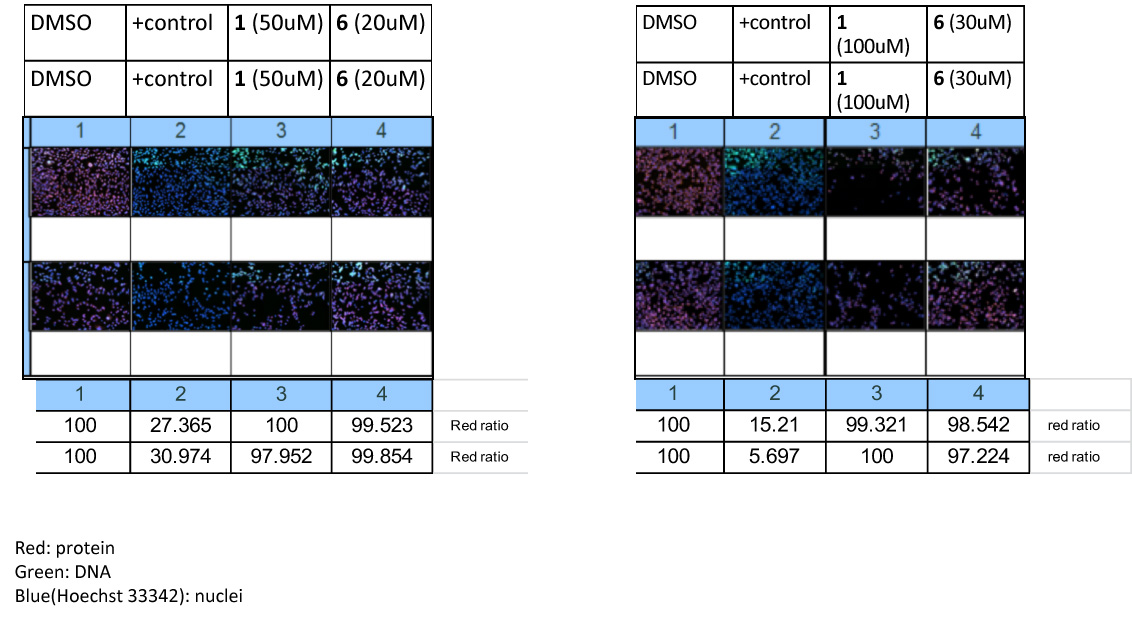


**Figure S4.** Effects of GA-01 on MDA-MB-231 *de novo* protein synthesis.


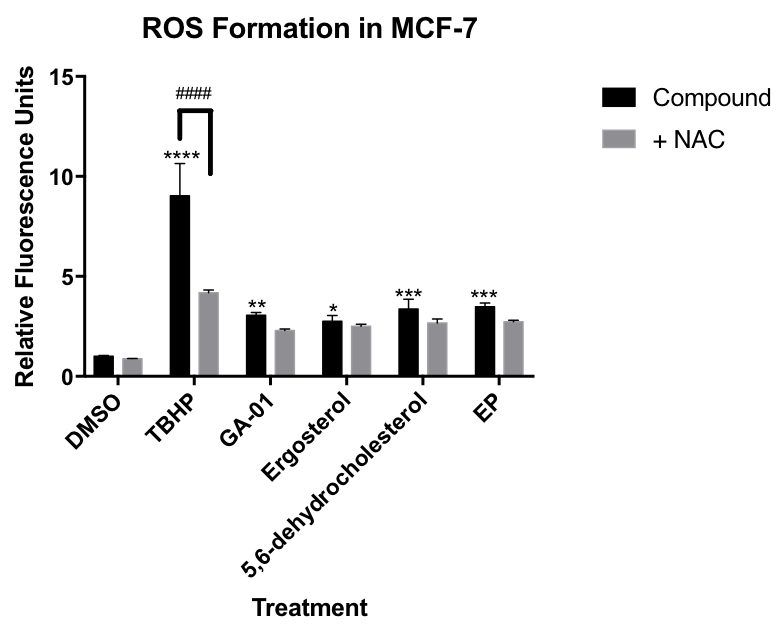


**Figure S5.** Reactive oxygen species (ROS) formation in MCF-7 cells. MCF-7 breast cancer cells were treated as described in the materials and methods section with GA-01 (50 μM), ergosterol (50 μM), 5, 6-dihydroergosterol (50 μM), EP (20 μM), or TBHP (100 μM) as a positive control. N-Acetyl Cysteine (100 μM) was added to inhibit ROS formation. Bars depict mean ± SEM of triplicates. ****P<0.0001, ***P<0.001, **P<0.01, *P<0.05 compared to vehicle. ^###^P<0.0001 compared to NAC.


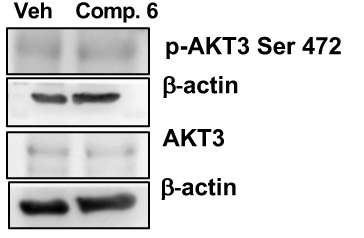


**Veh**

**EP**

**Figure S6.** EP does not affect AKT3 in SUM-149 cells. Western blot presented here were cropped to improve clarity. The entire blots can be found below (**Figure S7**).


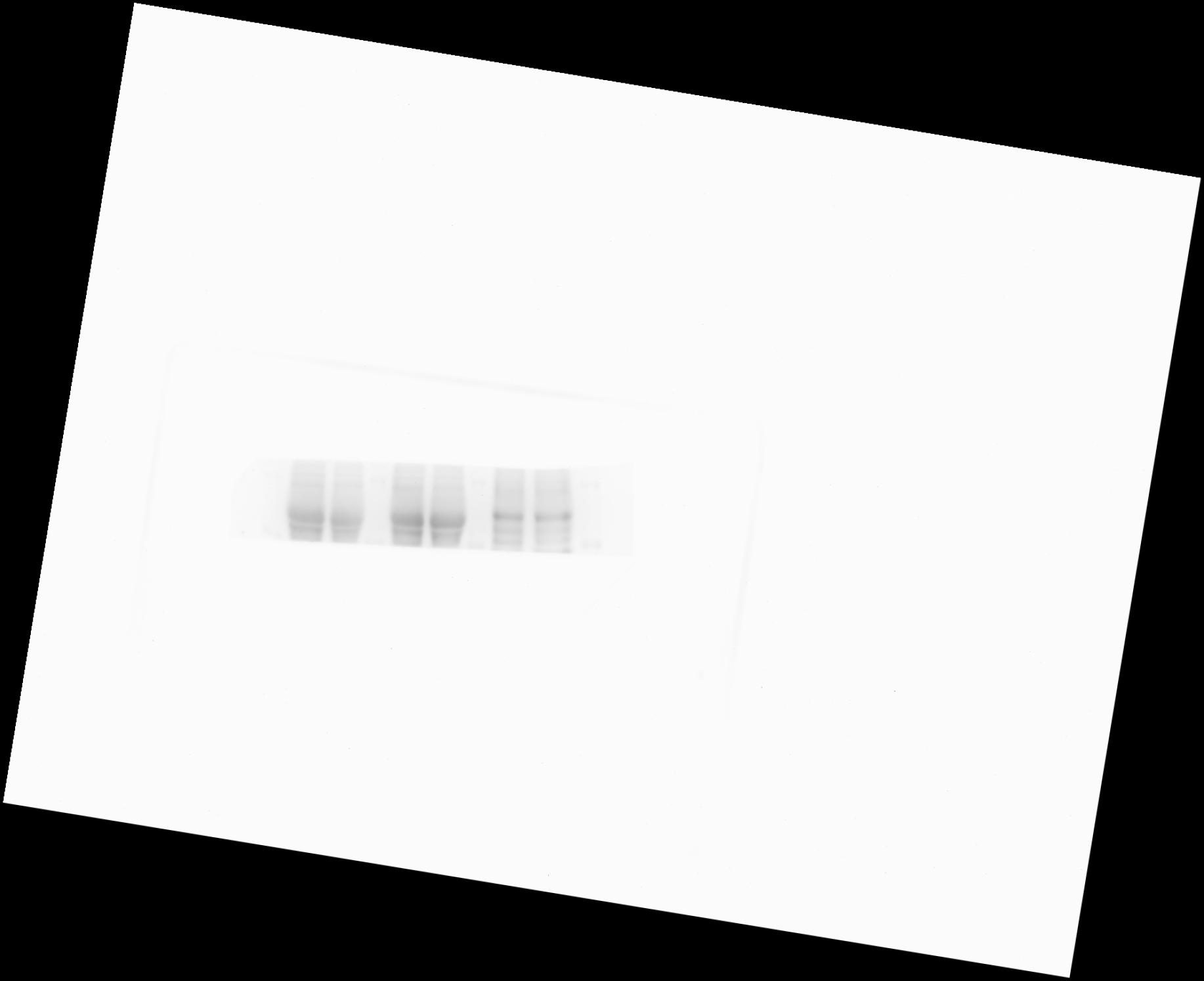


**Figure S7a.** AKT3.


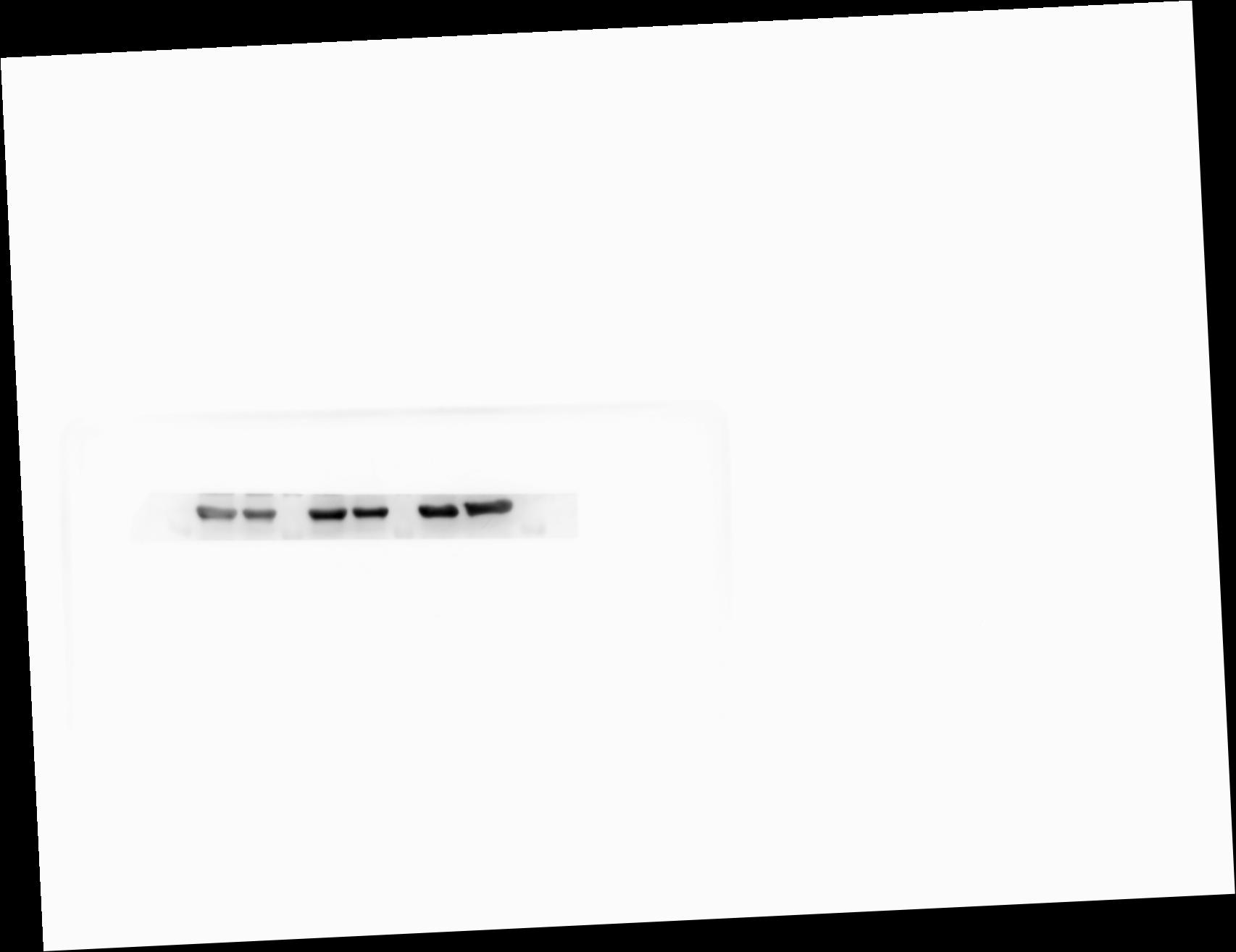


**Figure S7b.** β-actin representing the loading control of AKT3 (Figure S6a).


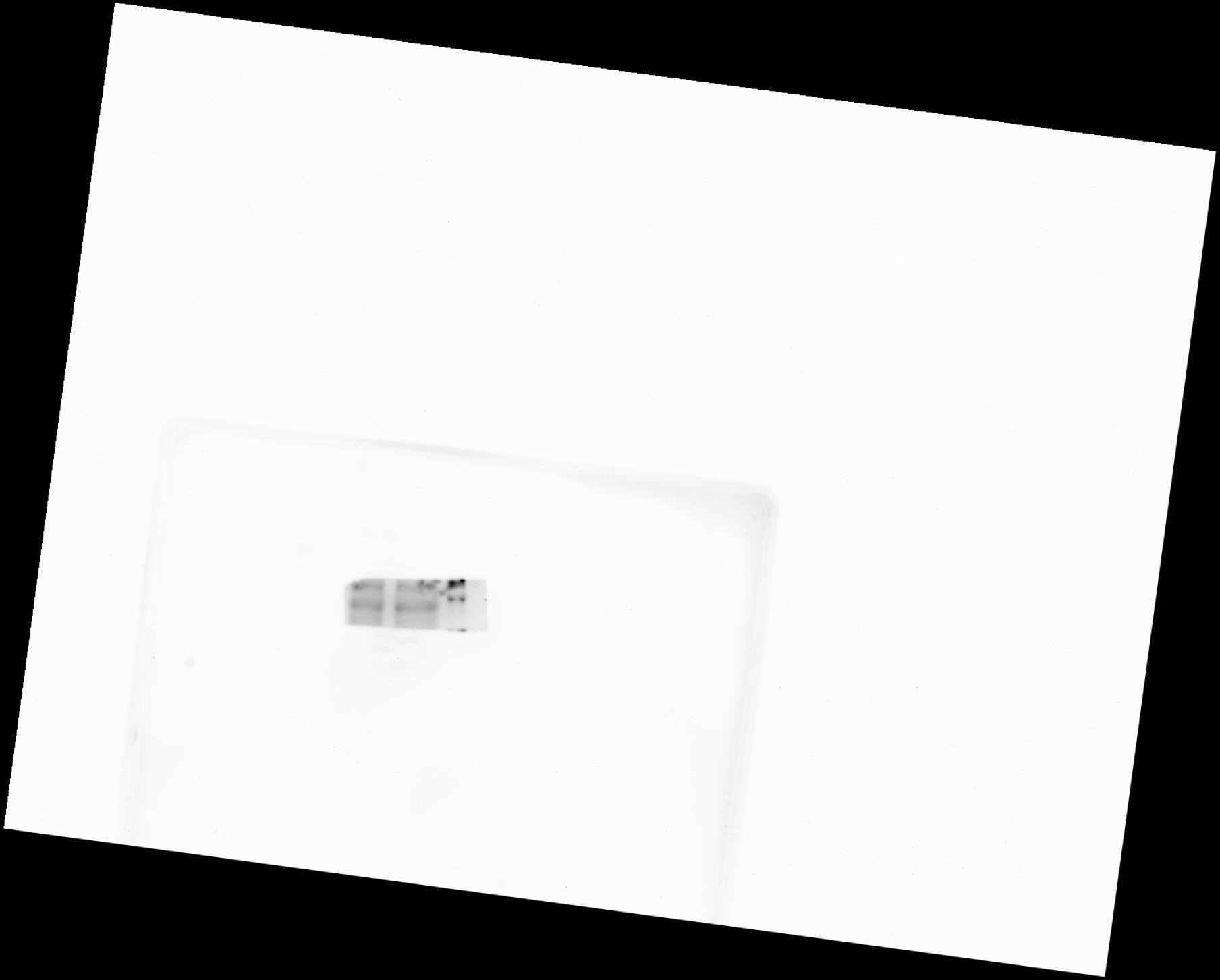


**Figure S7c.** p-AKT3 Ser 472.


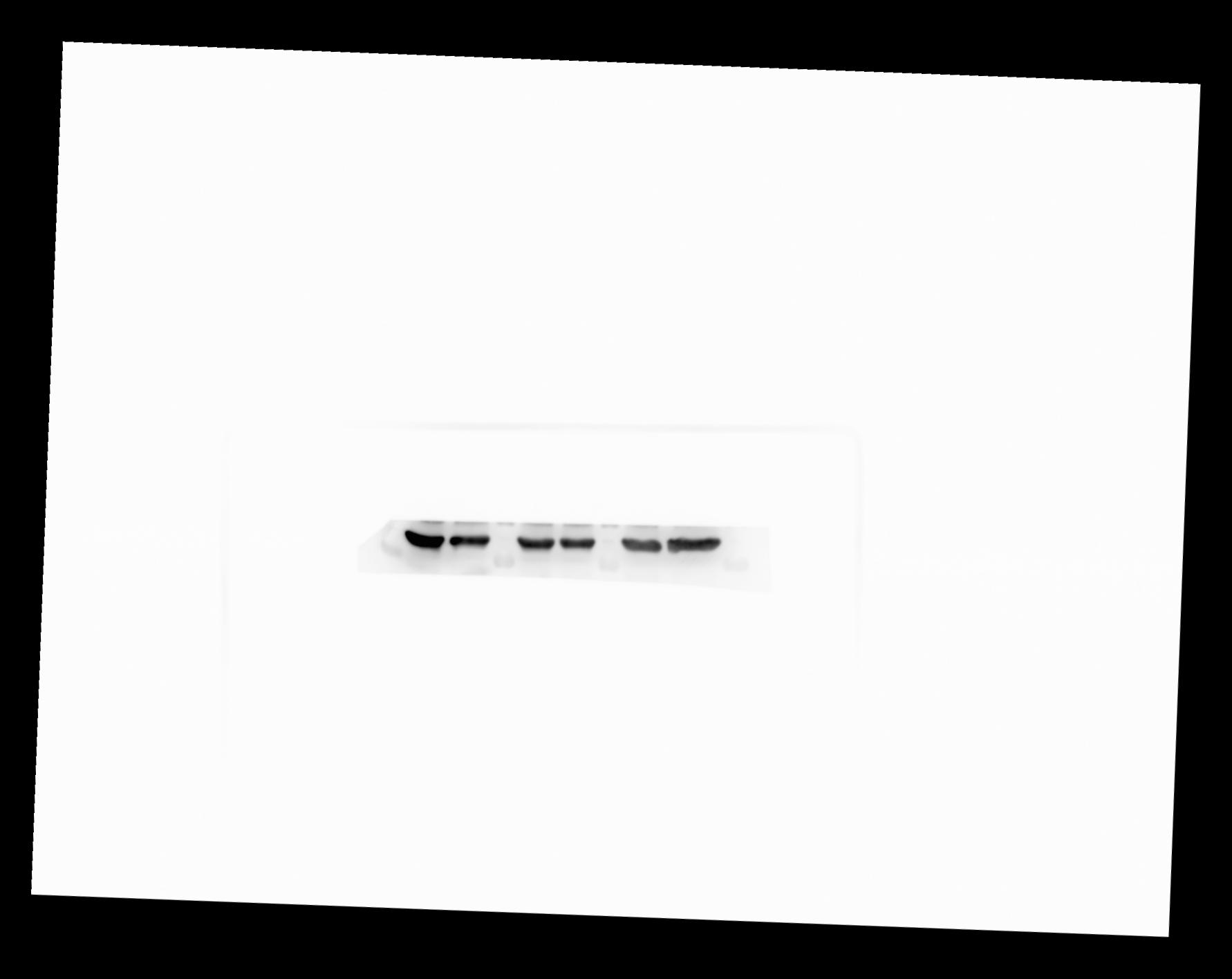


**Figure S7d.** β-actin representing the loading control of p-AKT3 Ser 472.


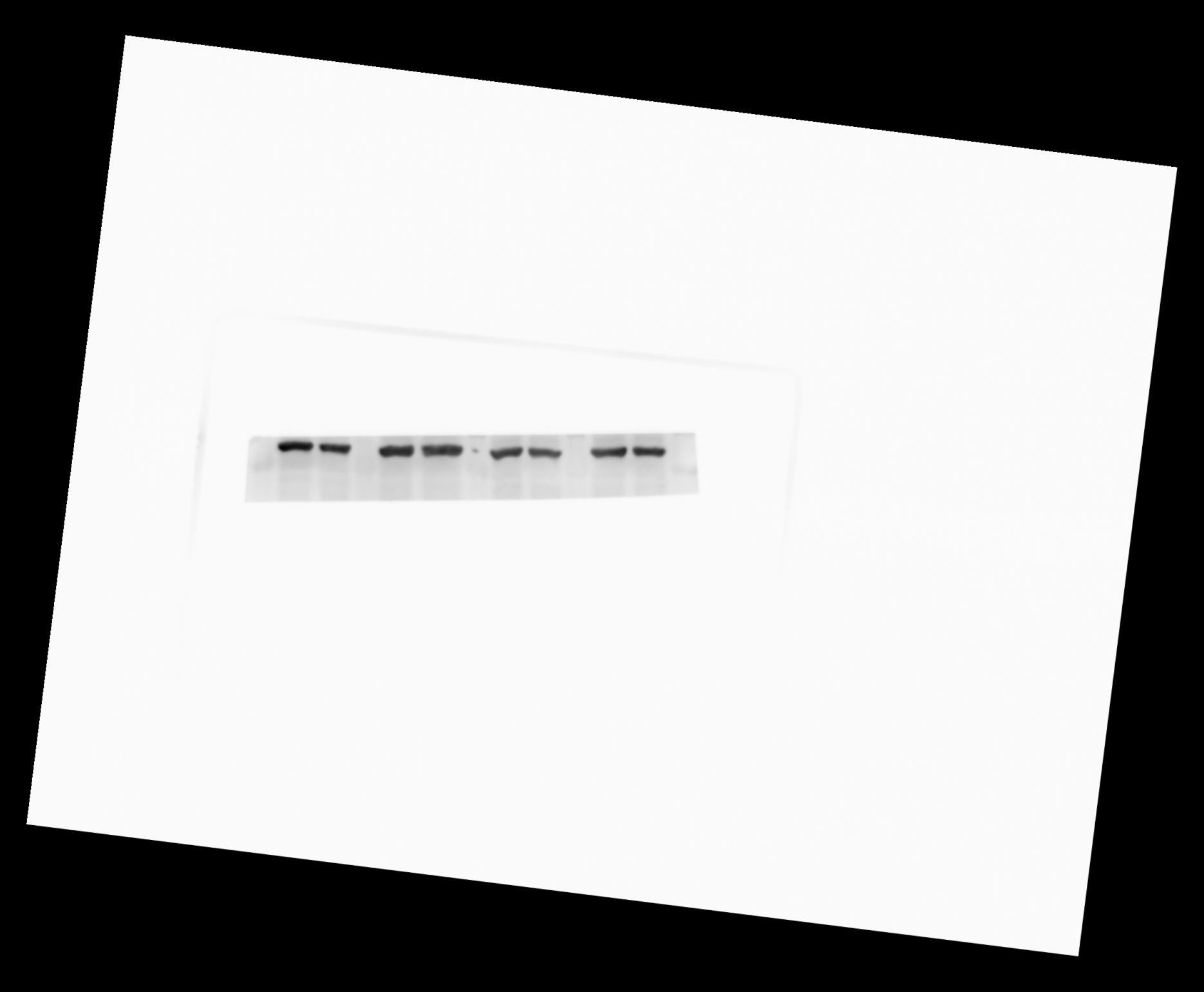


**Figure S7e.** β-actin representing the loading control of AKT.


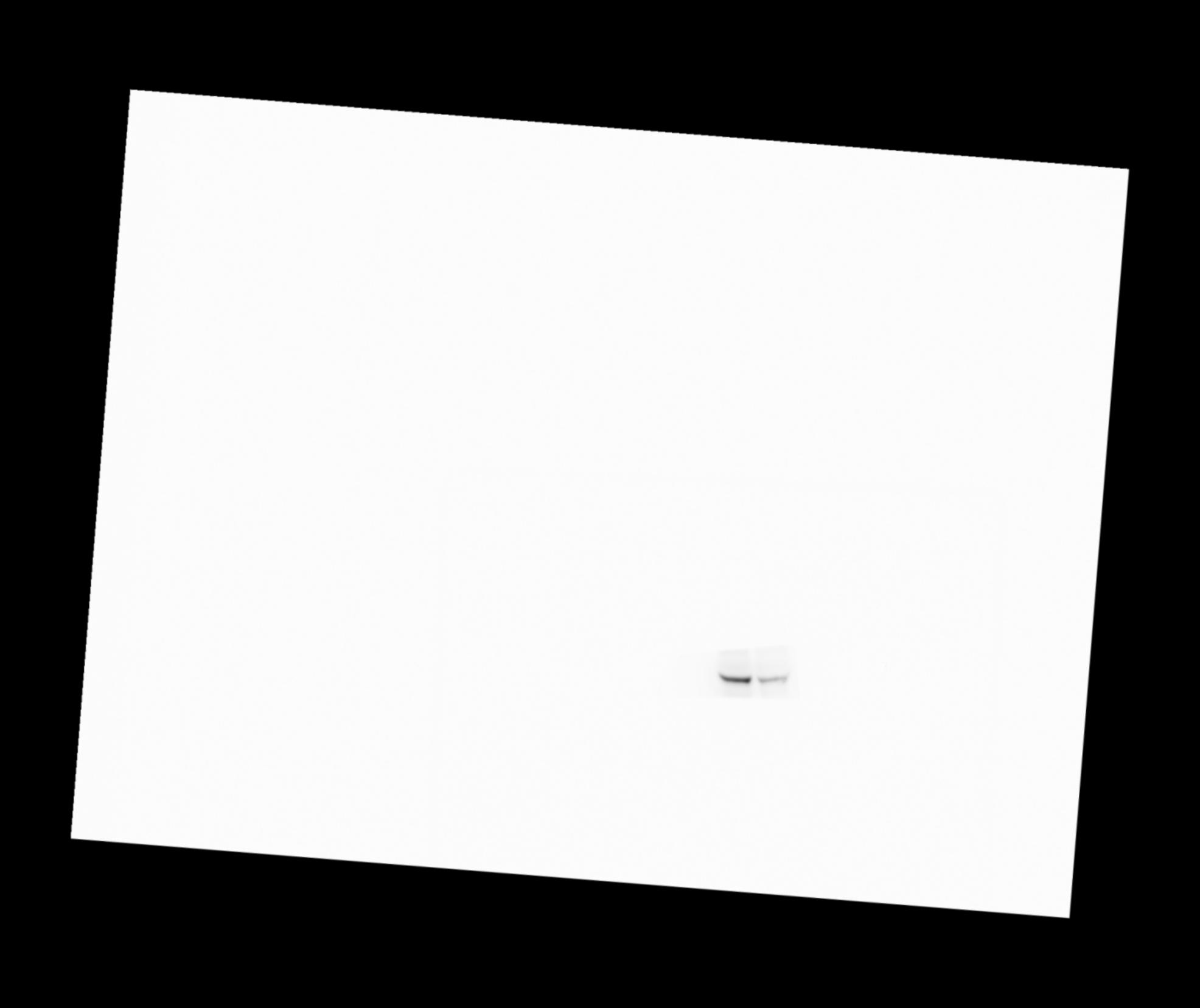


**Figure S7f.** AKT.


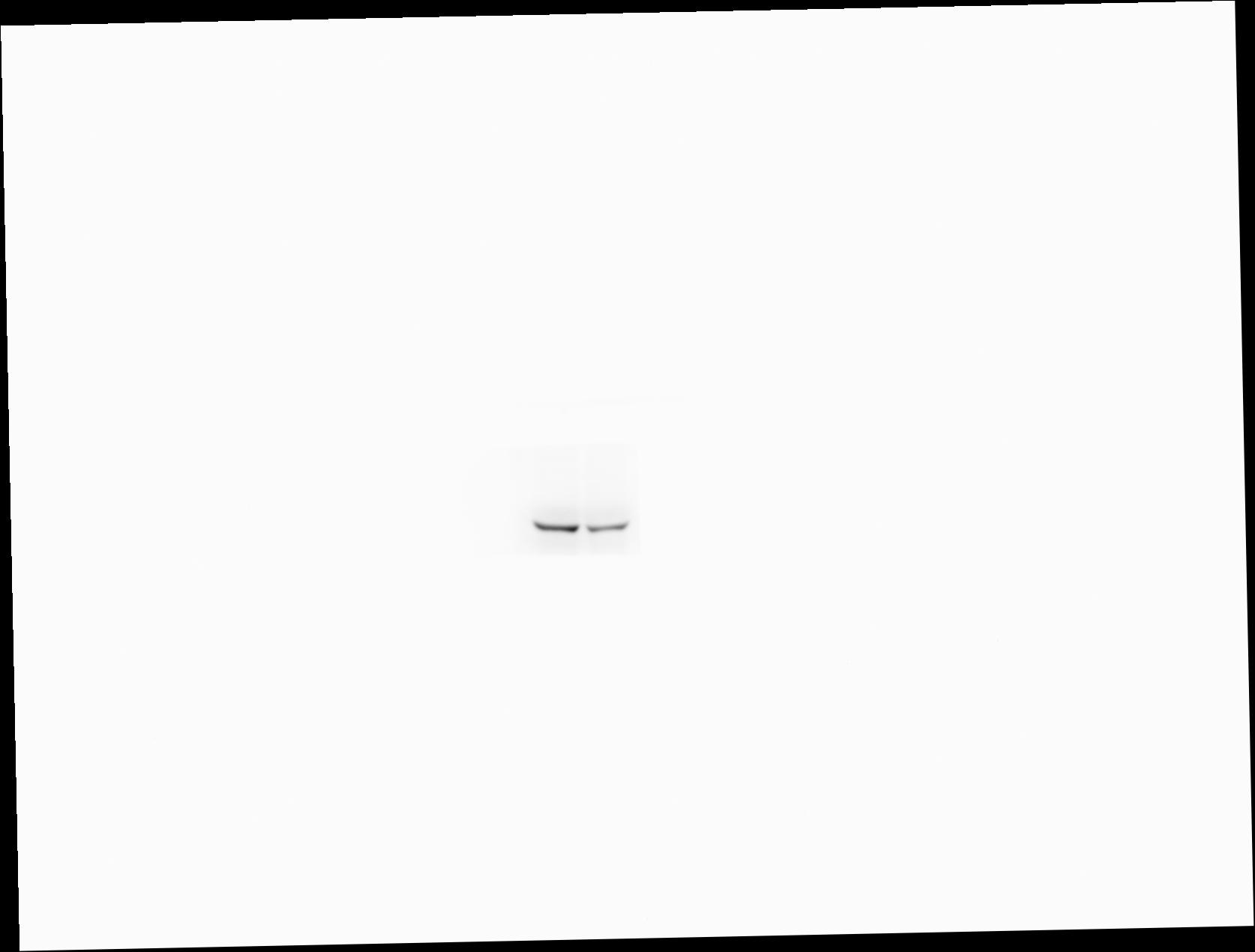


**Figure S7g.** p-AKT Ser 473.


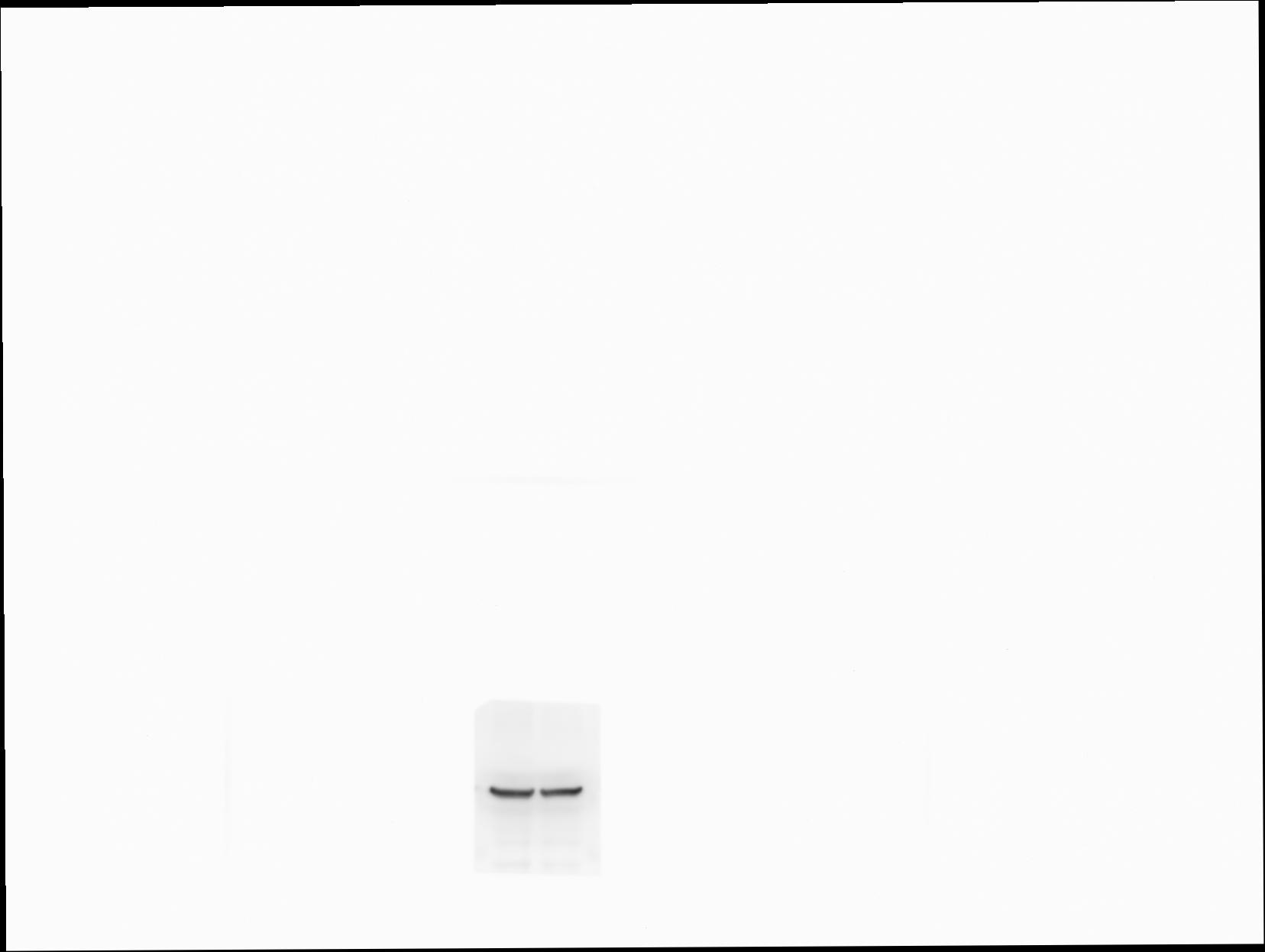


**Figure S7h.** β-tubulin representing the loading control of p-AKT Ser 473.


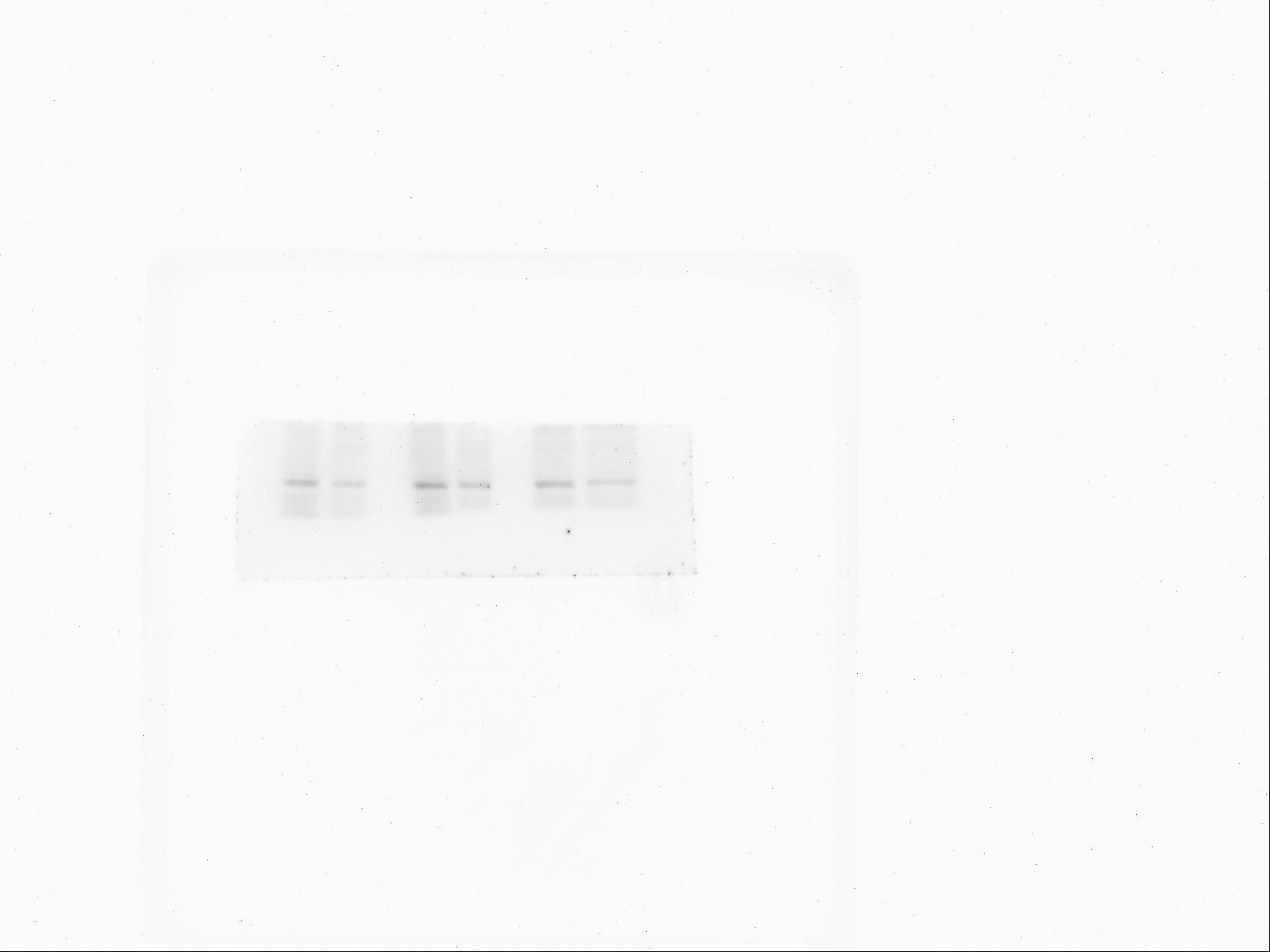


**Figure S7i.** BCL-2.


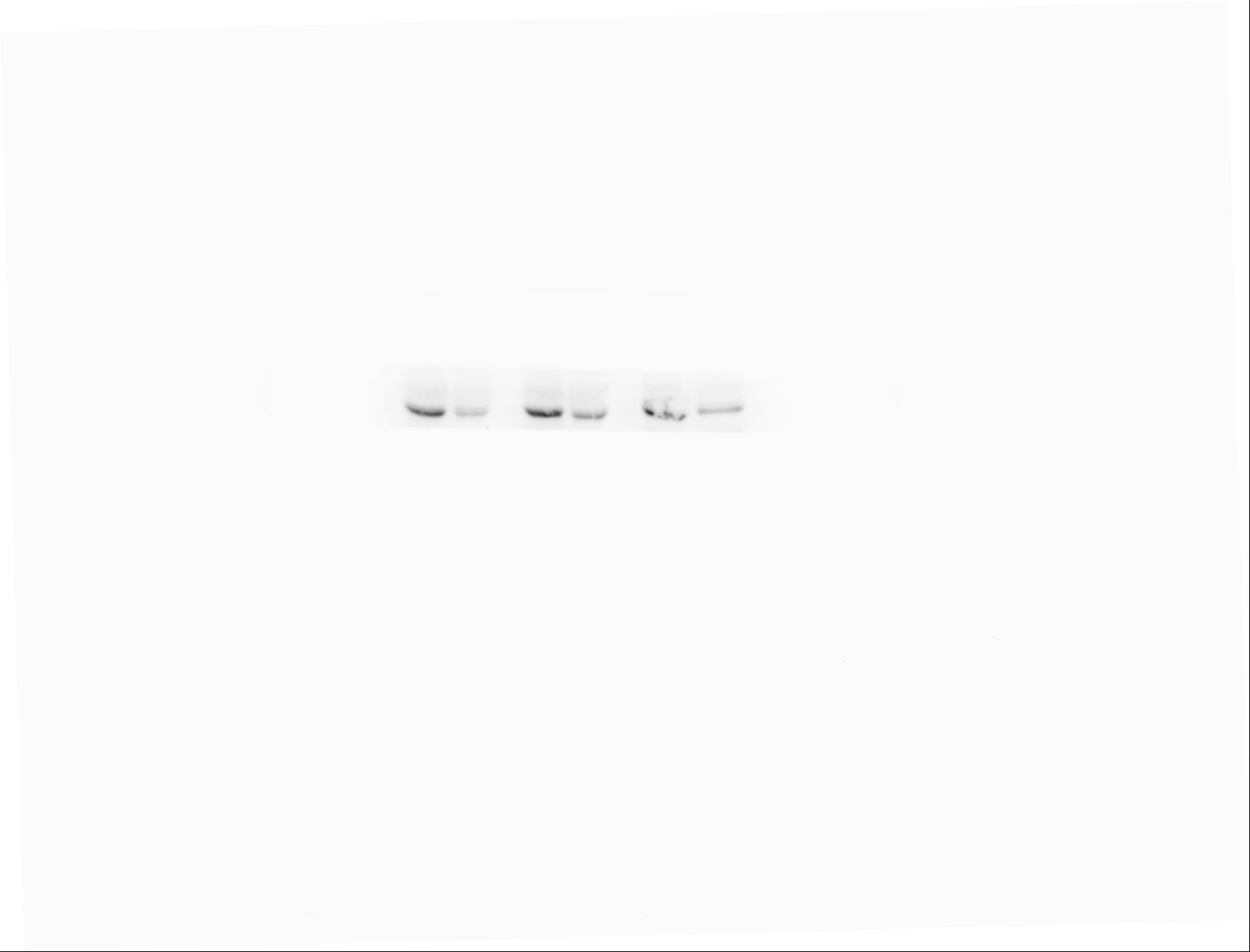


**Figure S7j.** AKT2.


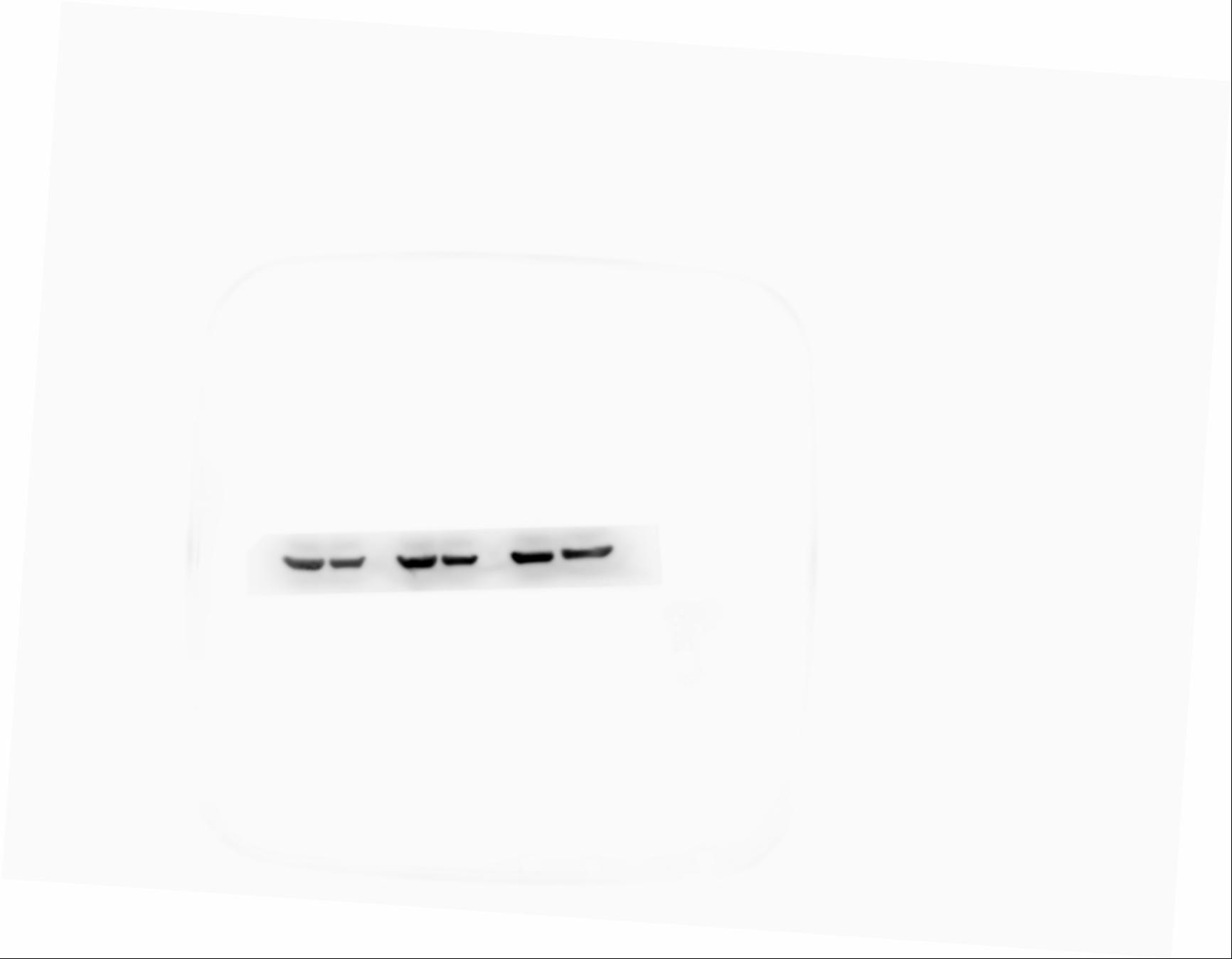


**Figure S7k.** β-actin representing the loading control of BCL-2 and AKT2.


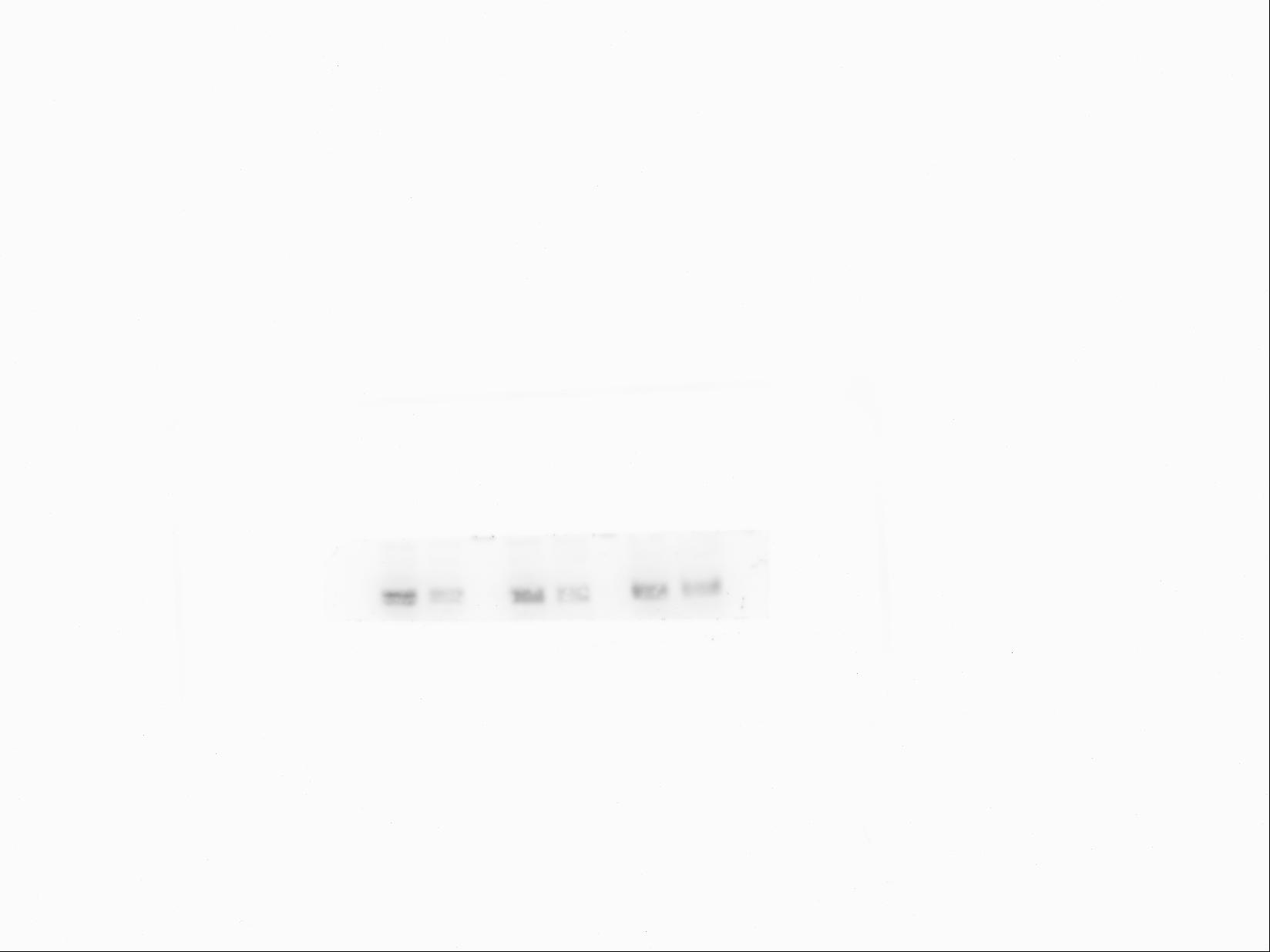


**Figure S7l.** BCL-XL.


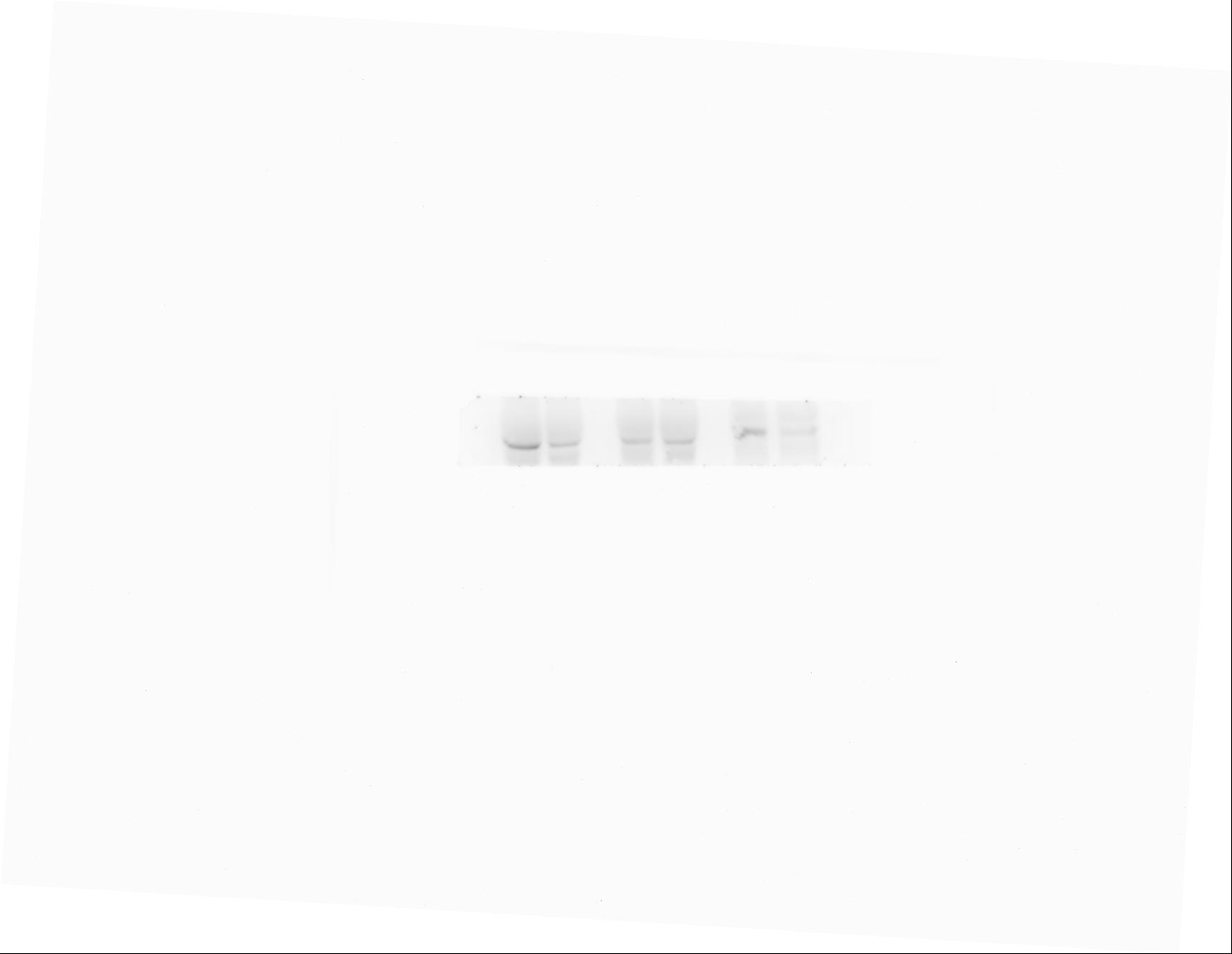


**Figure S7m.** p-AKT2 Ser 474.


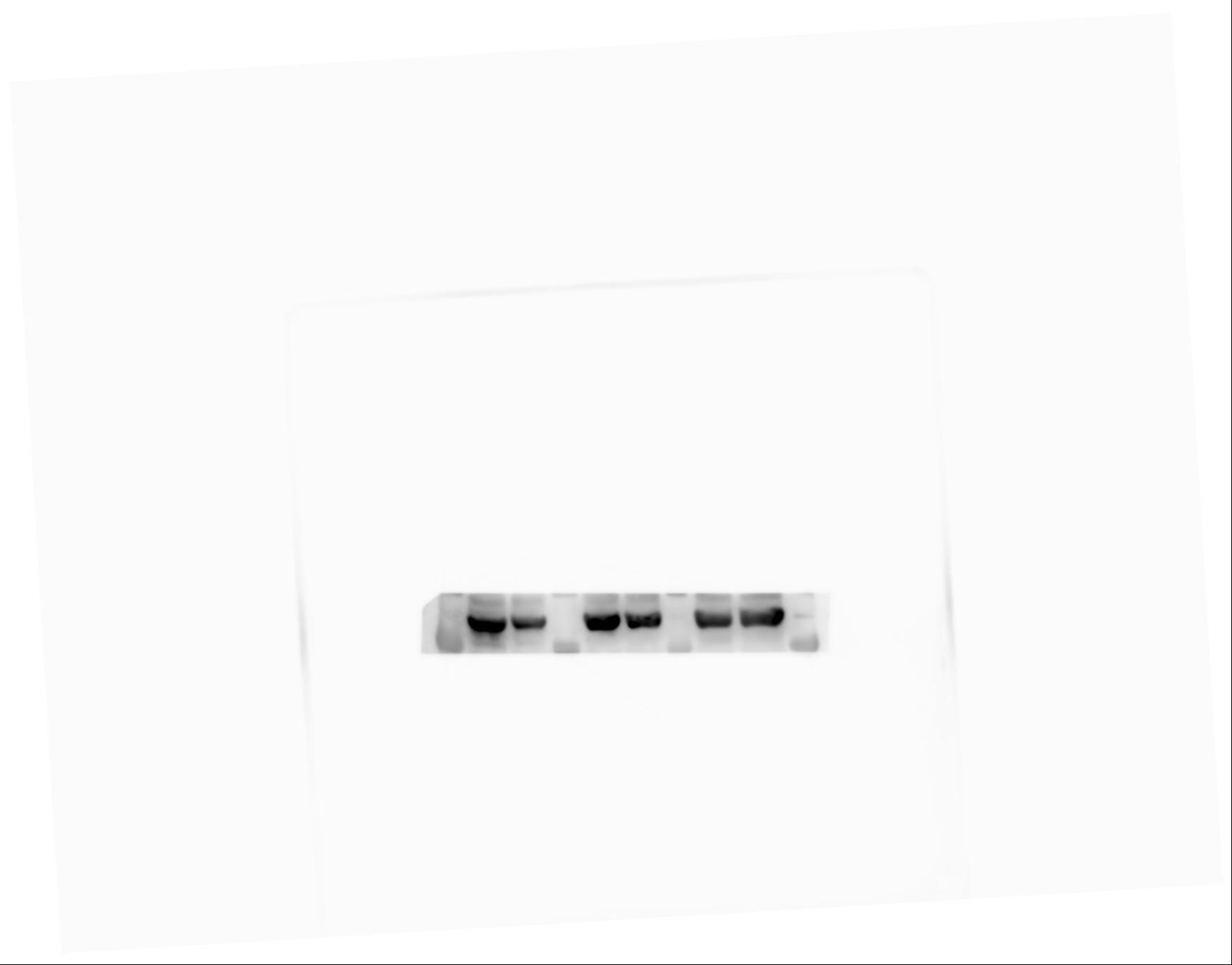


**Figure S7n.** β-actin representing the loading control of BCL-XL and p-AKT2 Ser 474.


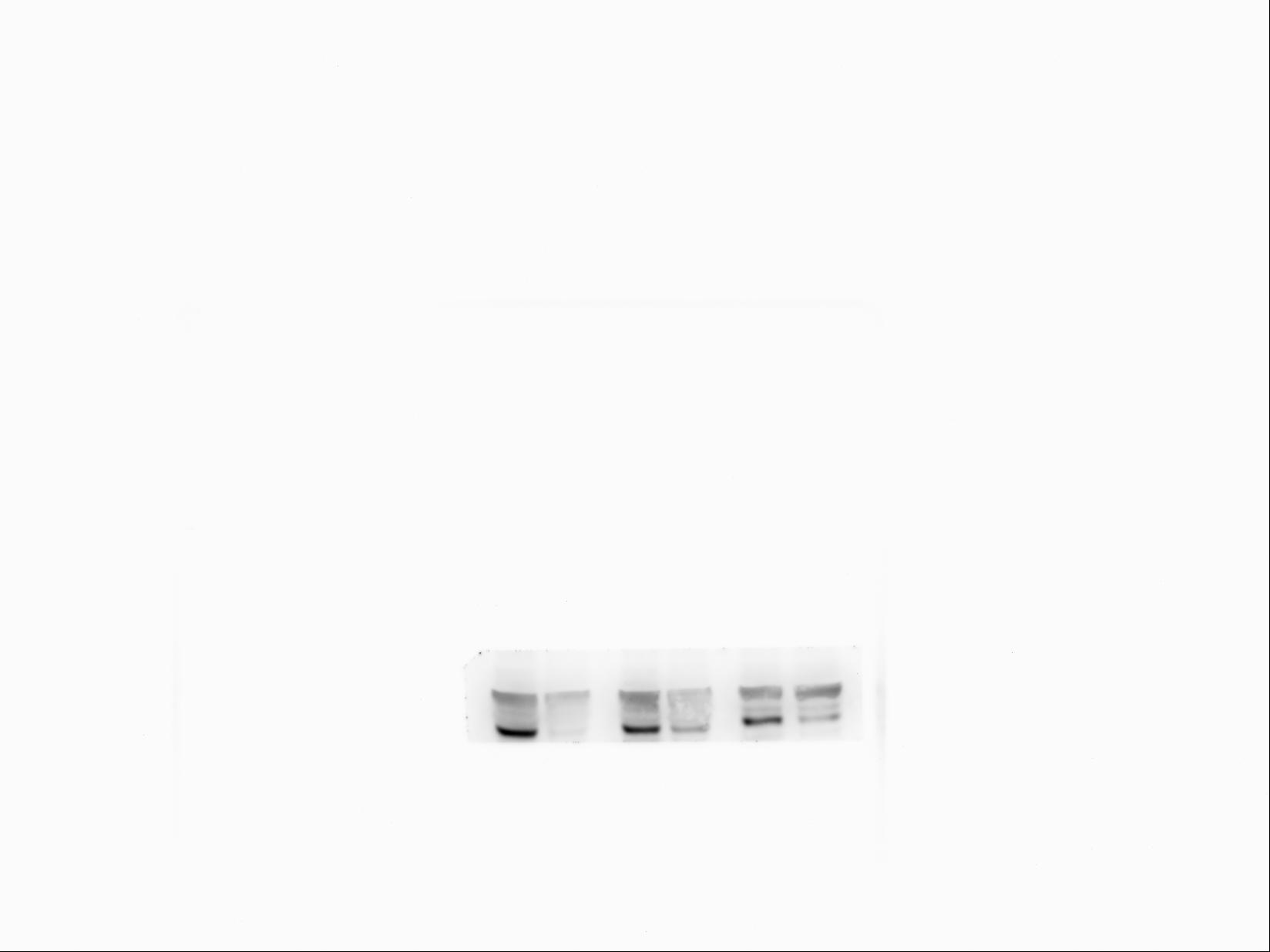


**Figure S7o.** c-Myc.


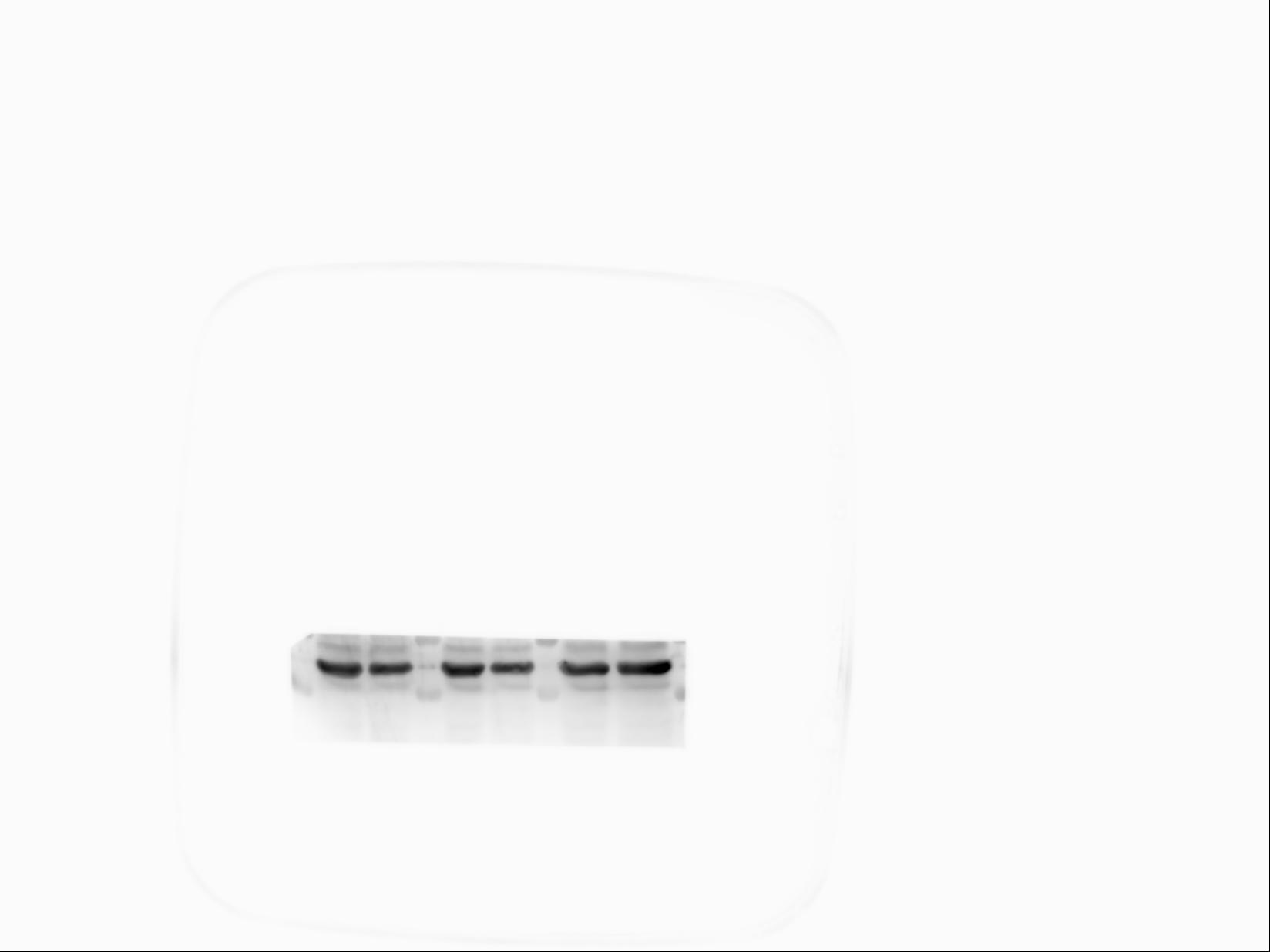


**Figure S7p.** β-actin representing the loading control of c-Myc.


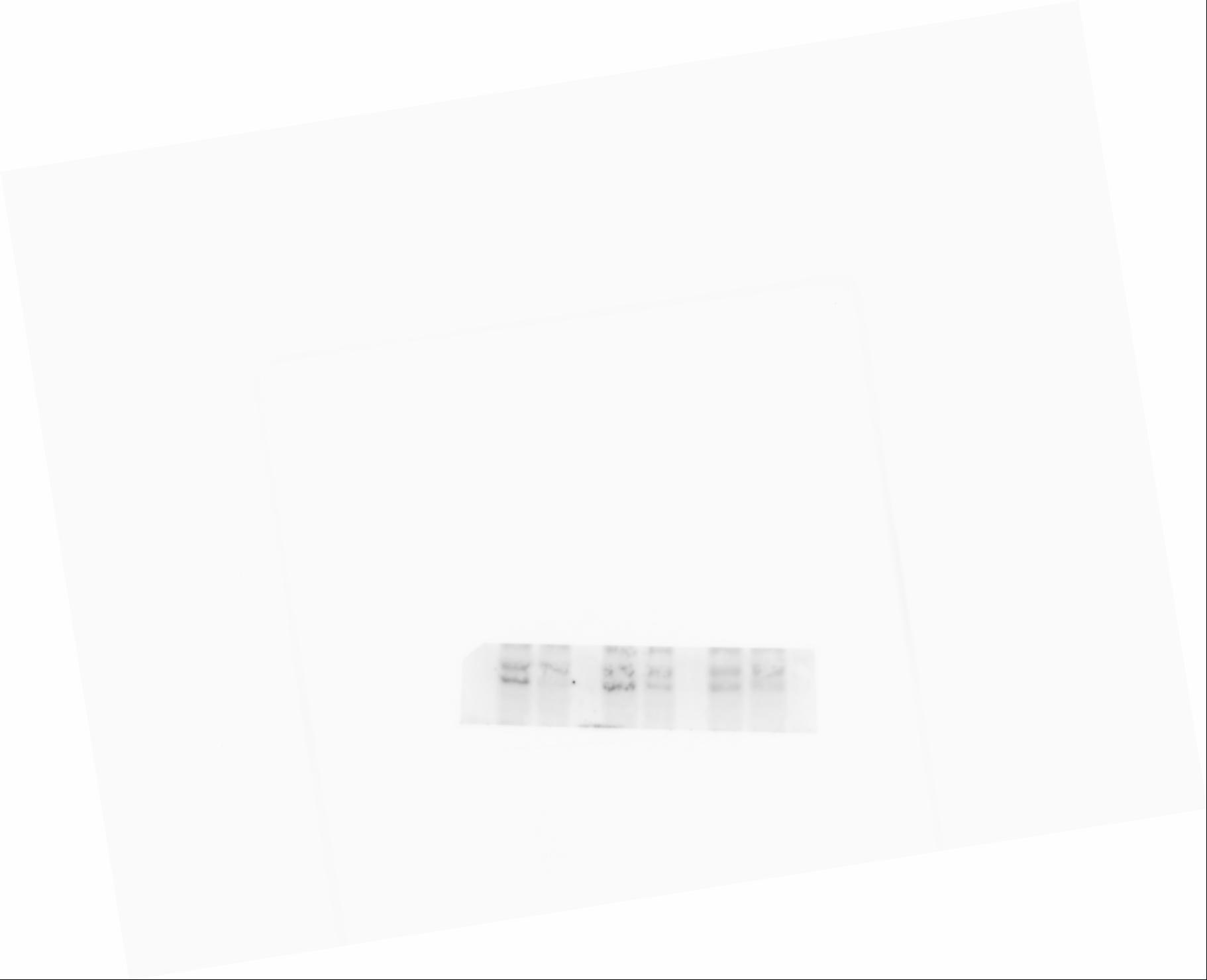


**Figure S7q.** Cyclin D1.


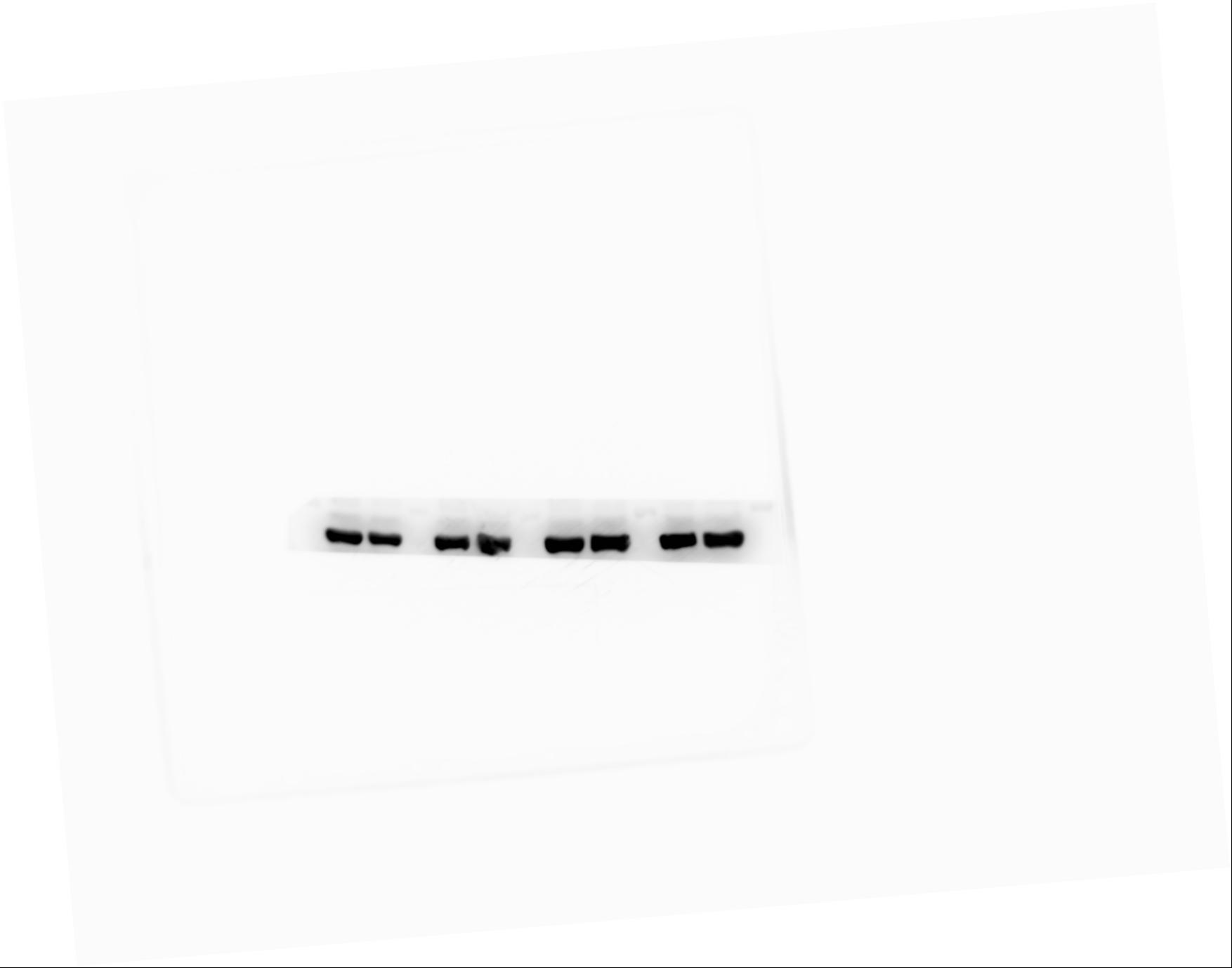


**Figure S7r.** β-actin representing the loading control of Cyclin D1.


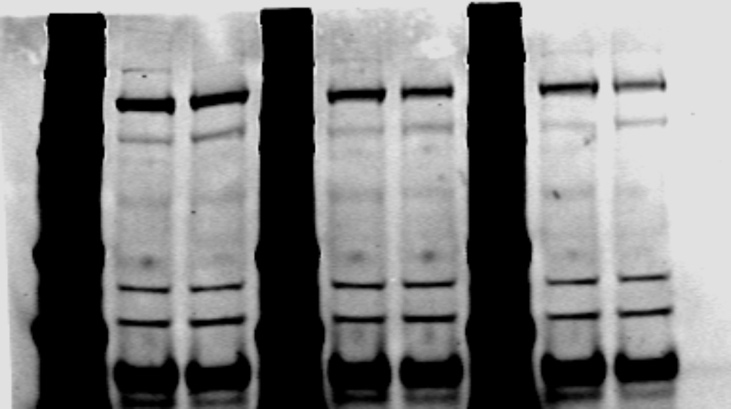


**Figure S7s.** PARP, cleaved PARP and Tubulin taken with the Lycor system.

b) X-Ray data for crystal structures of ergosterol (compound **4**) and 5,6-dehydroergosterol (compound **5**)

Fraction 98-99 precipitated, and crystals were collected, and single crystal diffraction studies were conducted on a Bruker Kappa APEX-II CCD diffractometer equipped with Cu K_α_ radiation (λ = 1.5478). Crystals of the subject compound were grown by dissolving approximately 1 mg of sample in 350 µL of Ethyl Acetate, which was then vapor diffused with Pentane over several days. A 0.114 × 0.085 × 0.076 mm piece of a colorless block was mounted on a Cryoloop with Paratone oil. Data were collected in a nitrogen gas stream at 100(2) K using φ and ϖ scans. Crystal-to-detector distance was 40 mm using variable exposure time (10s-60s) depending on θ, with a scan width of 1.0°. Data collection was 96.2% complete to 67.614° in *θ* (0.83Å). A total of 37758 reflections were collected covering the indices, -12<=h<=12, -8<=k<=8, -40<=l<=40. 8970 reflections were found to be symmetry independent, with a R_int_ of 0.0582. Indexing and unit cell refinement indicated a primitive, monoclinic lattice. The space group was found to be *P*2_1_. The data were integrated by using the Bruker SAINT software program and scaled by using the SADABS software program. Solution by direct methods (SHELXT) produced a complete phasing model consistent with the proposed structure.

All nonhydrogen atoms were refined anisotropically by full-matrix least-squares (SHELXL-2014). All hydrogen atoms were placed using a riding model. Their positions were constrained relative to their parent atom by using the appropriate HFIX command in SHELXL-2014. The absolute stereochemistry of the molecule was established by anomalous dispersion by using the Parson’s method with a Flack parameter of 0.002(230). Crystallographic data are summarized in Table 1, and detailed parameters are described in **Table S1-S6**. A ball and stick model is shown in Figure S8.

**
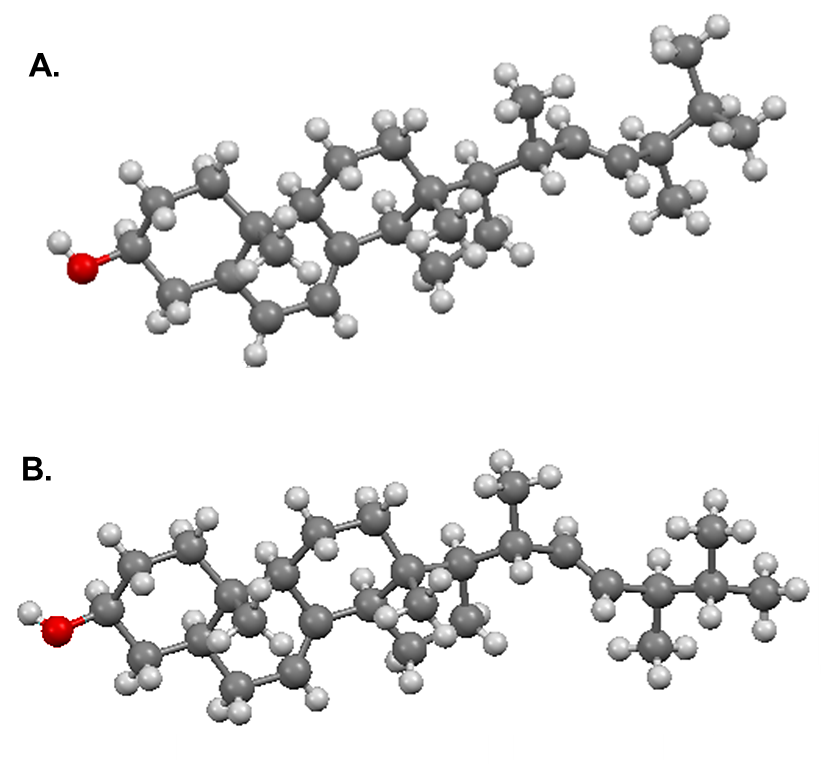
**

**Figure S8.** Ball and stick model of ergosterol (A) and 5,6-dehydroergosterol (B).

**Table S1.** Crystal data and structure refinement for ergosterol and 5,6-dehydroergosterol

**Table S2.** Atomic coordinates (× 10^4^) and equivalent isotropic displacement parameters (Å^2^× 10^3^) for CCDC 1833346. U(eq) is defined as one-third of the trace of the orthogonalized Uij tensor.

______________________________________________________________________________

x y z U(eq)

________________________________________________________________________________

O(1) 2772(13) 9880(16) 4762(2) 193(6)

C(1) 2356(13) 8471(14) 4139(2) 112(4)

C(2) 1877(15) 9869(17) 4412(2) 135(5)

C(3) 1880(13) 11672(16) 4227(2) 122(4)

C(4) 966(10) 11631(12) 3852(2) 90(3)

C(5) 1412(7) 10276(9) 3552(2) 59(2)

C(6) 1551(10) 8433(11) 3749(2) 88(3)

C(7) 1708(9) 6976(11) 3514(2) 84(2)

C(8) 971(7) 6952(9) 3126(2) 64(2)

C(9) 335(6) 8398(8) 2986(2) 47(1)

C(10) 313(6) 10110(9) 3213(2) 54(1)

C(11) 215(7) 11788(8) 2955(2) 55(1)

C(12) -719(7) 11624(8) 2594(2) 55(1)

C(13) -427(5) 9969(7) 2353(2) 42(1)

C(14) -580(5) 8356(7) 2622(2) 44(1)

C(15) -580(6) 6748(7) 2347(2) 53(1)

C(16) -1283(6) 7456(8) 1966(2) 52(1)

C(17) -1477(5) 9482(7) 2020(2) 44(1)

C(18) -1537(6) 10472(8) 1626(2) 52(1)

C(19) -2702(6) 9780(9) 1383(2) 55(1)

C(20) -2753(6) 9007(9) 1047(2) 55(1)

C(21) -3966(6) 8285(9) 824(2) 55(1)

C(22) -4404(6) 9477(9) 474(2) 60(2)

C(23) -3299(7) 9743(13) 198(2) 76(2)

C(24) -4951(8) 11257(12) 601(2) 81(2)

C(25) 2738(8) 10822(13) 3391(2) 84(2)

C(26) 944(5) 10085(8) 2197(2) 52(1)

C(27) -1619(7) 12517(8) 1660(2) 59(2)

C(28) -3795(7) 6349(10) 720(2) 73(2)

O(1') 8841(7) 6555(8) 4739(1) 101(2)

C(1') 7657(9) 7627(9) 4173(2) 75(2)

C(2') 8644(10) 6271(11) 4324(2) 80(2)

C(3') 8147(9) 4400(10) 4238(2) 78(2)

C(4') 7860(8) 4180(9) 3793(2) 64(2)

C(5') 6779(6) 5475(7) 3630(2) 46(1)

C(6') 7255(8) 7362(8) 3738(2) 64(2)

C(7') 6352(8) 8781(8) 3585(2) 64(2)

C(8') 5861(6) 8471(8) 3171(2) 54(1)

C(9') 5965(5) 6936(6) 2983(1) 36(1)

C(10') 6670(5) 5329(7) 3176(1) 40(1)

C(11') 6089(6) 3555(7) 3028(2) 43(1)

C(12') 5751(6) 3474(7) 2587(2) 44(1)

C(13') 4815(4) 5002(6) 2452(1) 32(1)

C(14') 5596(5) 6722(6) 2560(1) 34(1)

C(15') 4819(5) 8197(6) 2341(2) 43(1)

C(16') 4252(5) 7289(6) 1965(2) 42(1)

C(17') 4547(4) 5252(6) 2006(1) 33(1)

C(18') 3458(5) 4103(6) 1793(1) 34(1)

C(19') 3134(5) 4741(6) 1385(1) 35(1)

C(20') 1979(5) 5392(7) 1256(1) 41(1)

C(21') 1576(5) 5969(8) 847(2) 46(1)

C(22') 275(6) 5042(9) 695(2) 53(1)

C(23') -169(7) 5661(10) 283(2) 62(2)

C(24') 424(7) 3025(9) 709(2) 65(2)

C(25') 5452(7) 5091(12) 3797(2) 74(2)

C(26') 3523(5) 4925(8) 2652(1) 45(1)

C(27') 3817(6) 2126(7) 1786(2) 46(1)

C(28') 1466(8) 8026(9) 844(2) 68(2)

O(1S) 1212(10) 5128(11) 4993(2) 131(3)

O(2S) 3880(20) 6490(20) 5052(4) 291(10)

_________________________________________________________________________

**Table S3.**  Bond lengths [Å] and angles [°] for CCDC 1833346.

O(1)-C(2) 1.460(13)

C(1)-C(2) 1.506(13)

C(1)-C(6) 1.526(12)

C(2)-C(3) 1.492(17)

C(3)-C(4) 1.538(11)

C(4)-C(5) 1.536(10)

C(5)-C(6) 1.539(10)

C(5)-C(10) 1.563(8)

C(5)-C(25) 1.542(11)

C(6)-C(7) 1.374(11)

C(7)-C(8) 1.485(10)

C(8)-C(9) 1.334(8)

C(9)-C(10) 1.502(9)

C(9)-C(14) 1.509(8)

C(10)-C(11) 1.539(8)

C(11)-C(12) 1.515(8)

C(12)-C(13) 1.530(8)

C(13)-C(14) 1.534(7)

C(13)-C(17) 1.557(7)

C(13)-C(26) 1.525(7)

C(14)-C(15) 1.531(8)

C(15)-C(16) 1.546(9)

C(16)-C(17) 1.541(8)

C(17)-C(18) 1.543(8)

C(18)-C(19) 1.493(9)

C(18)-C(27) 1.537(9)

C(19)-C(20) 1.289(8)

C(20)-C(21) 1.505(8)

C(21)-C(22) 1.543(9)

C(21)-C(28) 1.505(10)

C(22)-C(23) 1.529(9)

C(22)-C(24) 1.518(11)

O(1')-C(2') 1.448(8)

C(1')-C(2') 1.493(11)

C(1')-C(6') 1.540(8)

C(2')-C(3') 1.510(11)

C(3')-C(4') 1.549(8)

C(4')-C(5') 1.539(8)

C(5')-C(6') 1.529(8)

C(5')-C(10') 1.563(7)

C(5')-C(25') 1.525(9)

C(6')-C(7') 1.475(9)

C(7')-C(8') 1.496(8)

C(8')-C(9') 1.325(7)

C(9')-C(10') 1.528(6)

C(9')-C(14') 1.488(7)

C(10')-C(11') 1.526(7)

C(11')-C(12') 1.535(7)

C(12')-C(13') 1.537(6)

C(13')-C(14') 1.543(6)

C(13')-C(17') 1.553(6)

C(13')-C(26') 1.518(6)

C(14')-C(15') 1.526(6)

C(15')-C(16') 1.538(7)

C(16')-C(17') 1.557(7)

C(17')-C(18') 1.545(6)

C(18')-C(19') 1.501(6)

C(18')-C(27') 1.523(7)

C(19')-C(20') 1.318(7)

C(20')-C(21') 1.501(7)

C(21')-C(22') 1.550(8)

C(21')-C(28') 1.543(8)

C(22')-C(23') 1.532(8)

C(22')-C(24') 1.517(9)

C(2)-C(1)-C(6) 112.8(8)

O(1)-C(2)-C(1) 108.1(9)

O(1)-C(2)-C(3) 109.2(12)

C(3)-C(2)-C(1) 110.6(8)

C(2)-C(3)-C(4) 108.8(10)

C(5)-C(4)-C(3) 113.0(7)

C(4)-C(5)-C(6) 108.5(6)

C(4)-C(5)-C(10) 109.2(5)

C(4)-C(5)-C(25) 111.7(7)

C(6)-C(5)-C(10) 107.3(6)

C(6)-C(5)-C(25) 109.9(6)

C(25)-C(5)-C(10) 110.2(5)

C(1)-C(6)-C(5) 113.5(8)

C(7)-C(6)-C(1) 117.3(7)

C(7)-C(6)-C(5) 117.5(6)

C(6)-C(7)-C(8) 117.9(6)

C(9)-C(8)-C(7) 121.2(6)

C(8)-C(9)-C(10) 121.7(6)

C(8)-C(9)-C(14) 122.7(6)

C(10)-C(9)-C(14) 114.9(5)

C(9)-C(10)-C(5) 115.0(5)

C(9)-C(10)-C(11) 113.3(4)

C(11)-C(10)-C(5) 112.5(5)

C(12)-C(11)-C(10) 114.9(5)

C(11)-C(12)-C(13) 112.1(5)

C(12)-C(13)-C(14) 106.2(4)

C(12)-C(13)-C(17) 116.2(4)

C(14)-C(13)-C(17) 99.8(4)

C(26)-C(13)-C(12) 111.0(5)

C(26)-C(13)-C(14) 112.9(4)

C(26)-C(13)-C(17) 110.2(4)

C(9)-C(14)-C(13) 113.8(4)

C(9)-C(14)-C(15) 120.3(5)

C(15)-C(14)-C(13) 104.0(4)

C(14)-C(15)-C(16) 103.7(4)

C(17)-C(16)-C(15) 106.8(5)

C(16)-C(17)-C(13) 103.5(4)

C(16)-C(17)-C(18) 111.3(5)

C(18)-C(17)-C(13) 121.5(5)

C(19)-C(18)-C(17) 108.1(5)

C(19)-C(18)-C(27) 110.1(5)

C(27)-C(18)-C(17) 114.1(5)

C(20)-C(19)-C(18) 129.8(6)

C(19)-C(20)-C(21) 126.9(6)

C(20)-C(21)-C(22) 112.0(5)

C(28)-C(21)-C(20) 111.4(6)

C(28)-C(21)-C(22) 113.6(5)

C(23)-C(22)-C(21) 112.2(6)

C(24)-C(22)-C(21) 111.9(6)

C(24)-C(22)-C(23) 111.2(7)

C(2')-C(1')-C(6') 112.3(6)

O(1')-C(2')-C(1') 106.9(7)

O(1')-C(2')-C(3') 110.7(6)

C(1')-C(2')-C(3') 110.8(6)

C(2')-C(3')-C(4') 109.3(6)

C(5')-C(4')-C(3') 112.1(5)

C(4')-C(5')-C(10') 108.7(4)

C(6')-C(5')-C(4') 106.8(5)

C(6')-C(5')-C(10') 107.9(4)

C(25')-C(5')-C(4') 111.6(6)

C(25')-C(5')-C(6') 110.8(6)

C(25')-C(5')-C(10') 111.0(5)

C(5')-C(6')-C(1') 114.4(5)

C(7')-C(6')-C(1') 111.9(6)

C(7')-C(6')-C(5') 113.6(5)

C(6')-C(7')-C(8') 112.8(5)

C(9')-C(8')-C(7') 124.5(5)

C(8')-C(9')-C(10') 121.3(5)

C(8')-C(9')-C(14') 123.2(4)

C(14')-C(9')-C(10') 114.9(4)

C(9')-C(10')-C(5') 112.3(4)

C(11')-C(10')-C(5') 113.3(4)

C(11')-C(10')-C(9') 112.4(4)

C(10')-C(11')-C(12') 114.9(4)

C(11')-C(12')-C(13') 111.3(4)

C(12')-C(13')-C(14') 104.6(4)

C(12')-C(13')-C(17') 116.9(4)

C(14')-C(13')-C(17') 101.0(4)

C(26')-C(13')-C(12') 111.6(4)

C(26')-C(13')-C(14') 111.4(4)

C(26')-C(13')-C(17') 110.6(4)

C(9')-C(14')-C(13') 114.7(4)

C(9')-C(14')-C(15') 119.7(4)

C(15')-C(14')-C(13') 104.1(4)

C(14')-C(15')-C(16') 104.4(4)

C(15')-C(16')-C(17') 107.3(4)

C(13')-C(17')-C(16') 103.1(4)

C(18')-C(17')-C(13') 118.4(4)

C(18')-C(17')-C(16') 112.0(4)

C(19')-C(18')-C(17') 111.9(4)

C(19')-C(18')-C(27') 109.3(4)

C(27')-C(18')-C(17') 112.6(4)

C(20')-C(19')-C(18') 124.6(4)

C(19')-C(20')-C(21') 127.1(5)

C(20')-C(21')-C(22') 111.1(5)

C(20')-C(21')-C(28') 108.0(5)

C(28')-C(21')-C(22') 112.6(5)

C(23')-C(22')-C(21') 111.6(5)

C(24')-C(22')-C(21') 110.7(5)

C(24')-C(22')-C(23') 110.6(5)

_____________________________________________________________ **Table S4.** Anisotropic displacement parameters (Å^2^× 10^3^) for CCDC 1833346.

The anisotropic displacement factor exponent takes the form: -2π2[ h^2^ a*^2^U^11^ + ... + 2 h k a* b* U^12^ ]

______________________________________________________________________________

U^11^ U^22^ U^33^ U^23^ U^13^ U^12^

______________________________________________________________________________

O(1) 324(14) 196(10) 54(3) -10(5) -33(5) 136(10)

C(1) 177(10) 107(7) 52(4) 17(4) 5(5) 62(7)

C(2) 229(13) 131(9) 44(4) 0(5) -2(6) 73(10)

C(3) 178(11) 120(8) 63(5) -20(5) -30(6) 59(8)

C(4) 139(8) 75(5) 55(4) -8(4) -10(4) 22(5)

C(5) 72(4) 55(4) 50(3) 6(3) 4(3) 18(3)

C(6) 145(8) 70(5) 49(4) 18(3) 15(4) 36(5)

C(7) 99(6) 67(5) 85(5) 12(4) -3(4) 39(4)

C(8) 73(4) 44(3) 75(4) -1(3) 15(3) 19(3)

C(9) 50(3) 41(3) 53(3) 10(2) 18(2) 9(2)

C(10) 61(3) 49(3) 52(3) 4(3) 10(3) 2(3)

C(11) 71(4) 36(3) 57(3) 0(2) -3(3) 8(3)

C(12) 70(4) 38(3) 56(3) 1(2) -3(3) 5(3)

C(13) 34(2) 41(3) 54(3) 0(2) 11(2) 9(2)

C(14) 35(3) 35(2) 64(3) 2(2) 16(2) 2(2)

C(15) 56(3) 33(3) 70(4) -3(2) 16(3) -1(2)

C(16) 44(3) 51(3) 65(4) -6(3) 18(3) -4(2)

C(17) 40(3) 39(3) 54(3) -1(2) 9(2) -1(2)

C(18) 53(3) 53(3) 52(3) -1(2) 8(3) 10(3)

C(19) 52(3) 63(4) 51(3) -3(3) 8(3) 4(3)

C(20) 46(3) 64(4) 56(3) 2(3) 5(3) 6(3)

C(21) 41(3) 70(4) 55(3) -4(3) 3(2) -5(3)

C(22) 53(3) 67(4) 58(3) -7(3) 3(3) -8(3)

C(23) 62(4) 104(6) 61(4) 0(4) 3(3) -11(4)

C(24) 83(5) 79(5) 79(5) 0(4) -7(4) 10(4)

C(25) 72(5) 103(6) 75(5) 19(4) -13(4) 0(4)

C(26) 47(3) 52(3) 57(3) 8(3) 3(2) 0(3)

C(27) 68(4) 54(4) 55(3) 4(3) 2(3) 5(3)

C(28) 64(4) 67(4) 87(5) -3(4) -2(4) -9(3)

O(1') 174(6) 78(4) 47(2) -1(2) -37(3) -2(4)

C(1') 124(6) 51(4) 46(3) -3(3) -19(4) -3(4)

C(2') 122(6) 73(5) 40(3) 4(3) -24(4) -10(4)

C(3') 123(7) 64(4) 43(3) 3(3) -22(4) 14(4)

C(4') 95(5) 51(3) 42(3) 0(3) -18(3) 12(3)

C(5') 67(3) 36(3) 35(3) 2(2) 0(2) 3(2)

C(6') 97(5) 49(4) 44(3) -2(3) -18(3) 7(3)

C(7') 92(5) 44(3) 54(4) -3(3) -11(3) 4(3)

C(8') 73(4) 34(3) 53(3) 0(2) -12(3) 12(3)

C(9') 36(2) 27(2) 45(3) 3(2) -1(2) 4(2)

C(10') 47(3) 36(3) 36(2) 4(2) 2(2) 3(2)

C(11') 54(3) 31(2) 43(3) 4(2) -4(2) 10(2)

C(12') 55(3) 33(2) 42(3) 1(2) -6(2) 16(2)

C(13') 33(2) 26(2) 39(2) -1(2) 4(2) 4(2)

C(14') 31(2) 28(2) 41(2) 3(2) 2(2) 3(2)

C(15') 48(3) 27(2) 52(3) 5(2) -6(2) -2(2)

C(16') 48(3) 33(3) 45(3) 4(2) -4(2) 5(2)

C(17') 30(2) 31(2) 40(2) 7(2) 4(2) 4(2)

C(18') 30(2) 35(2) 37(2) 1(2) 4(2) 1(2)

C(19') 38(2) 29(2) 37(2) -1(2) 4(2) 2(2)

C(20') 44(3) 41(3) 40(3) -2(2) 4(2) 4(2)

C(21') 38(3) 54(3) 46(3) 4(2) 3(2) 13(2)

C(22') 53(3) 66(4) 41(3) 1(3) 0(2) 11(3)

C(23') 57(4) 78(4) 51(3) 2(3) -6(3) 11(3)

C(24') 79(4) 60(4) 53(3) -5(3) -15(3) -1(3)

C(25') 93(5) 89(5) 42(3) 2(3) 13(3) -17(4)

C(26') 39(2) 58(3) 37(2) 4(2) 6(2) -10(2)

C(27') 57(3) 28(2) 52(3) -1(2) -8(2) 9(2)

C(28') 83(5) 52(4) 67(4) 11(3) -16(4) 4(3)

O(1S) 200(8) 98(5) 86(4) 27(4) -45(5) -8(5)

O(2S) 450(30) 182(13) 221(13) 86(12) -87(16) -85(16)

______________________________________________________________________________ **Table S5.** Hydrogen coordinates (× 10^4^) and isotropic displacement parameters (Å^2^× 10 ^3^)

for CCDC 1833346.

________________________________________________________________________________

x y z U(eq)

________________________________________________________________________________

H(1) 2509 10633 4920 290

H(1A) 2307 7283 4264 135

H(1B) 3294 8709 4094 135

H(2) 961 9567 4483 162

H(3A) 2790 11995 4165 147

H(3B) 1563 12580 4408 147

H(4A) 57 11327 3920 108

H(4B) 939 12837 3734 108

H(7) 2272 6014 3596 101

H(8) 951 5886 2976 77

H(10) -538 10076 3344 64

H(11C) -78 12802 3112 66

H(11D) 1109 12078 2873 66

H(12C) -641 12705 2432 66

H(12D) -1641 11560 2673 66

H(14) -1497 8433 2712 53

H(15C) -1072 5731 2452 63

H(15D) 334 6360 2305 63

H(16C) -735 7225 1743 63

H(16D) -2148 6857 1915 63

H(17) -2363 9639 2127 53

H(18) -720 10169 1492 63

H(19) -3532 9934 1492 66

H(20) -1938 8880 928 66

H(21) -4697 8329 1006 66

H(22) -5141 8838 324 71

H(23D) -2570 10410 331 114

H(23E) -3641 10415 -32 114

H(23F) -2976 8576 116 114

H(24D) -5666 11048 774 122

H(24E) -5297 11926 371 122

H(24F) -4245 11947 739 122

H(25D) 2902 10077 3165 126

H(25E) 3456 10655 3592 126

H(25F) 2698 12081 3312 126

H(26D) 1617 10111 2415 78

H(26E) 1007 11176 2042 78

H(26F) 1090 9040 2033 78

H(27D) -2361 12839 1815 88

H(27E) -1754 13042 1400 88

H(27F) -793 12974 1787 88

H(28D) -4617 5899 589 109

H(28E) -3584 5654 957 109

H(28F) -3073 6235 545 109

H(1') 9638 6365 4811 152

H(1'A) 8037 8838 4213 90

H(1'B) 6858 7546 4323 90

H(2') 9498 6461 4200 96

H(3'A) 7329 4182 4373 93

H(3'B) 8821 3518 4333 93

H(4'A) 8683 4399 3660 77

H(4'B) 7575 2937 3736 77

H(6') 8087 7530 3601 77

H(7'A) 6819 9943 3603 77

H(7'B) 5585 8852 3748 77

H(8') 5445 9441 3033 65

H(10') 7598 5364 3094 48

H(11A) 5275 3311 3163 51

H(11B) 6730 2591 3100 51

H(12A) 5326 2313 2520 52

H(12B) 6576 3555 2449 52

H(14') 6450 6616 2432 40

H(15A) 5407 9203 2280 51

H(15B) 4100 8653 2495 51

H(16A) 4674 7781 1737 51

H(16B) 3286 7496 1930 51

H(17') 5394 5011 1882 40

H(18') 2639 4223 1938 41

H(19') 3810 4671 1207 42

H(20') 1330 5509 1441 50

H(21') 2291 5615 676 55

H(22') -431 5382 871 64

H(23A) -333 6952 284 94

H(23B) -984 5034 195 94

H(23C) 525 5392 107 94

H(24A) 1077 2651 528 98

H(24B) -430 2464 635 98

H(24C) 720 2656 974 98

H(25A) 4751 5744 3647 112

H(25B) 5482 5475 4070 112

H(25C) 5269 3805 3782 112

H(26A) 3109 3754 2606 67

H(26B) 2927 5862 2545 67

H(26C) 3699 5111 2932 67

H(27A) 4689 1984 1682 69

H(27B) 3153 1480 1620 69

H(27C) 3838 1643 2051 69

H(28A) 703 8392 986 102

H(28B) 1350 8448 574 102

H(28C) 2274 8545 969 102

H(1SA) 2002 5604 5023 196

H(1SB) 1200 4375 5186 196

H(2SA) 4466 5615 5146 436

H(2SB) 4014 7288 5237 436

________________________________________________________________________________  **Table S6.** Hydrogen bonds for CCDC 1833346 [Å and °].

____________________________________________________________________________

D-H...A d(D-H) d(H...A) d(D...A) <(DHA)

____________________________________________________________________________

O(1)-H(1)...O(1')#1 0.84 1.98 2.750(12) 151.4

O(1')-H(1')...O(1S)#2 0.84 1.91 2.718(11) 160.3

O(1S)-H(1SA)...O(2S) 0.88 2.01 2.88(2) 173.7

O(1S)-H(1SB)...O(1')#3 0.87 2.13 2.830(10) 137.1

____________________________________________________________________________

Symmetry transformations used to generate equivalent atoms:

#1 -x+1,y+1/2,-z+1 #2 x+1,y,z #3 -x+1,y-1/2,-z+1

c) General experimental chemistry for compounds **1**-**7**.

**Ganoderic acid A (Compound 1, GA-01).** Extracted GA-01 was validated by comparison with previously reported NMR studies.[[1]](#footnote-1) The isopropanol extraction of the mushroom powder (1 g) of *Ganoderma lucidum* was concentrated and subjected to Biotage silica gel chromatography; the titled compound [(2R,6R)-6-((5R,7S,10S,13R,14R,15S,17R)-7,15-dihydroxy-4,4,10,13,14-pentamethyl-3,11-dioxo-2,3,4,5,6,7,10,11,12,13,14,15,16,17-tetradecahydro-1H-cyclopenta[a]phenanthren-17-yl)-2-methyl-4-oxoheptanoic acid] was isolated as clear liquid. ^1^H NMR (500 MHz, Chloroform-*d*) δ 4.78 (t, *J* = 8.0 Hz, 1H), 4.64 – 4.59 (m, 1H), 3.0-0.75 (m, 40H); ^13^C NMR (126 MHz, Acetone-d6) δ 216.22, 208.80, 199.88, 176.98, 160.99, 140.54, 55.48, 54.95, 52.69, 50.16, 49.17, 48.93, 47.52, 47.09, 46.97, 38.67, 36.35, 35.13, 34.69, 33.45, 31.99, 27.54, 20.98, 19.95, 19.72, 17.60, 17.41. ^13^C NMR (126 MHz, Pyr-*d5*) δ 216.40, 208.98, 199.71, 178.21, 161.42, 139.69, 71.98, 69.10, 68.46, 55.97, 54.50, 52.19, 49.67, 48.71, 48.38, 46.90, 46.55, 38.06, 36.64, 35.82, 35.38, 34.39, 32.85, 32.02, 29.86, 29.73, 29.38, 27.03, 20.59, 20.10, 19.50, 19.33, 17.42, 17.28. HRMS (ESI-TOF) calculated for C_30_H_45_O_7_ ([M + H]^+^): 517.3165, found: 517.3169.

**Ganoderic acid A, methyl ester (Compound 1a, GA-01-ME).** GA-01 (1.0 equiv) was treated with TMSCHN_2_ (1.0M in diethyl ether, 1.10 equiv) in anhydrous MeOH (0.5mL) and was stirred for 1 h at RT. The reaction mixture was then diluted with brine and extracted with EtOAc. The combined organic phase was dried over MgSO_4_ and concentrated under reduced pressure. The crude product was purified by silica gel column chromatography (Hex/EtOAc, 1:9) to provide GA-01-ME [methyl (2R,6R)-6-((5R,7S,10S,13R,14R,15S,17R)-7,15-dihydroxy-4,4,10,13,14-pentamethyl-3,11-dioxo-2,3,4,5,6,7,10,11,12,13,14,15,16,17-tetradecahydro-1H-cyclopenta[a]phenanthren-17-yl)-2-methyl-4-oxoheptanoate] (90% yield).[[2]](#footnote-2)

^1^H NMR (500 MHz, Chloroform-*d*) δ 4.83 – 4.77 (m, 1H), 4.65 – 4.59 (m, 1H), 3.67 (s, 3H), 2.94 (td, *J* = 7.8, 5.2 Hz, 1H), 2.87 – 2.79 (m, 2H), 2.74 (d, *J* = 15.7 Hz, 1H), 2.60-1.20 (m, 24H), 1.18 (d, *J* = 7.2 Hz, 3H), 1.11 (d, *J* = 9.0 Hz, 3H), 0.99 (s, 3H), 0.88 (d, *J* = 6.3 Hz, 3H); ^13^C NMR (126 MHz, CDCl_3_) δ 216.85, 208.39, 199.39, 176.22, 158.77, 140.34, 72.50, 68.91, 53.89, 53.41, 51.90, 51.70, 49.62, 48.73, 48.10, 46.78, 46.73, 46.59, 37.98, 36.46, 35.49, 34.60, 34.26, 32.58, 29.69, 29.16, 27.31, 20.69, 19.60, 19.49, 19.34, 17.25, 17.09. HRMS (ESI-TOF) calculated for C_31_H_47_O_7_ ([M + H]^+^): 531.3322, found: 531.3327.

**Ergosterol (Compound 4).** Extracted ergosterol was validated by comparison with previously reported NMR studies.[[3]](#footnote-3) The isopropanol extraction of the mushroom powder (1 g) of *Ganoderma lucidum* was concentrated and subjected to Biotage silica gel chromatography; the titled compound [(3S,9S,10R,13R,14R,17R)-17-((2R,5R,E)-5,6-dimethylhept-3-en-2-yl)-10,13-dimethyl-2,3,4,9,10,11,12,13,14,15,16,17-dodecahydro-1H-cyclopenta[a]phenanthren-3-ol] was isolated as a white solid. ^1^H NMR (500 MHz, Chloroform-*d*) δ 5.57 (dd, *J* = 5.7, 2.5 Hz, 1H), 5.39 (dd, *J* = 5.7, 2.9 Hz, 1H), 5.25-5.10 (m, 2H), 3.68-3.55 (m, 1H), 2.47 (ddd, *J* = 14.3, 4.8, 2.4 Hz, 1H), 2.32 – 2.22 (m, 1H), 2.10-1.10 (m, 19H), 1.04 (d, *J* = 6.7 Hz, 3H), 0.95 (s, 3H), 0.92 (d, *J* = 6.8 Hz, 3H), 0.85-0.80 (m, 6H), 0.63 (s, 3H); ^13^C NMR (126 MHz, CDCl_3_) δ 141.36, 139.77, 135.56, 131.96, 119.57, 116.27, 70.46, 55.72, 54.55, 46.24, 42.83, 42.81, 40.79, 40.42, 39.07, 38.37, 37.02, 33.08, 31.99, 28.29, 22.99, 21.10, 19.95, 19.64, 17.60, 16.28, 12.05. HRMS (ESI-TOF) calculated for C_28_H_45_O ([M + H]^+^): 397.3470, found: 397.3469.

**Ergosterol sulfonamide (Compound 4a).** A solution of ergosterol (1.0 equiv) and benzenesulfonyl isocyanate (1.10 equiv) in anhydrous THF was stirred at 25°C for 2 h until completion by TLC progress. The reaction mixture was then diluted with brine and extracted with EtOAc. The combined organic phase was dried over MgSO_4_ and concentrated under reduced pressure. The crude product was purified by silica gel column chromatography (Hex/EtOAc, 1:9) to provide ergosterol sulfonamide [(3S,9S,10R,13R,14R,17R)-17-((2R,5R,E)-5,6-dimethylhept-3-en-2-yl)-10,13-dimethyl-2,3,4,9,10,11,12,13,14,15,16,17-dodecahydro-1H-cyclopenta[a]phenanthren-3-yl (phenylsulfonyl)carbamate] (93% yield). ^1^H NMR (500 MHz, Chloroform-*d*) δ 8.06 – 8.03 (m, 2H), 7.66 (t, *J* = 7.5 Hz, 1H), 7.56 (t, *J* = 7.7 Hz, 2H), 5.52 (dd, *J* = 5.9, 2.4 Hz, 1H), 5.35 (dt, *J* = 5.7, 2.8 Hz, 1H), 5.23 – 5.16 (m, 2H), 4.59 (tt, *J* = 11.5, 4.5 Hz, 1H), 2.45 (ddd, *J* = 14.3, 5.1, 2.3 Hz, 1H), 2.28 (ddd, *J* = 14.3, 11.9, 3.9 Hz, 1H), 2.10-1.15 (m, 19H), 1.03 (d, *J* = 6.6 Hz, 3H), 0.95-0.80 (m, 12H), 0.61 (s, 3H); ^13^C NMR (126 MHz, CDCl_3_) δ 149.67, 141.75, 138.53, 137.48, 135.49, 133.93, 131.99, 128.95, 128.38, 120.64, 116.19, 76.31, 55.67, 54.49, 45.91, 42.79, 40.41, 38.94, 37.62, 36.94, 36.31, 33.07, 28.24, 27.88, 22.95, 21.09, 20.99, 19.94, 19.63, 17.59, 16.09, 12.05. HRMS (ESI-TOF) calculated for C_35_H_50_NO_4_S ([M + H]^+^): 580.3461, found: 580.3464.

**5,6-dihydroergosterol (Compound 5).** Extracted **5,6-dihydroergosterol** was validated by comparison with previously reported NMR studies.[[4]](#footnote-4)4 The isopropanol extraction of the mushroom powder (1g) of *Ganoderma lucidum* was concentrated and subjected to Biotage silica gel chromatography; the titled compound [(3S,5S,9R,10S,13R,14R,17R)-17-((2R,5S,E)-5,6-dimethylhept-3-en-2-yl)-10,13-dimethyl-2,3,4,5,6,9,10,11,12,13,14,15,16,17-tetradecahydro-1H-cyclopenta[a]phenanthren-3-ol] was isolated as a solid. ^1^H NMR (500 MHz, Chloroform-*d*) δ 5.18 (dd, *J* = 10.9, 7.3 Hz, 2H), 5.15 (bs, 1H), 3.59 (tt, *J* = 10.9, 4.5 Hz, 1H), 2.10-1.15 (m, 21H), 1.14 – 1.07 (m, 1H), 1.01 (d, *J* = 6.6 Hz, 3H), 0.91 (dd, *J* = 6.9, 2.4 Hz, 3H), 0.89-0.75 (m, 10H), 0.54 (s, 3H); ^13^C NMR (126 MHz, CDCl_3_) δ 139.54, 135.65, 131.86, 117.44, 71.05, 55.94, 55.09, 49.43, 42.80, 40.25, 37.97, 37.13, 33.08, 31.46, 29.62, 21.10, 19.94, 19.64, 17.59, 13.03, 12.08. HRMS (ESI-TOF) calculated for C_28_H_47_O ([M + H]^+^): 399.3627, found: 399.3631.

**5,6-dihydroergosterol** **sulfonamide (Compound 5a).** A solution of 5,6-dihydroergosterol (1.0 equiv) and benzenesulfonyl isocyanate (1.10 equiv) in dry THF was stirred at 25°C for 2 h until completion by TLC progress. The reaction mixture was then diluted with brine and extracted with EtOAc. The combined organic phase was dried over MgSO_4_ and concentrated under reduced pressure. The crude product was purified by silica gel column chromatography (Hex/EtOAc, 1:9) to provide 5,6-dihydroergosterol sulfonamide [(3S,5S,9R,10S,13R,14R,17R)-17-((2R,5S,E)-5,6-dimethylhept-3-en-2-yl)-10,13-dimethyl-2,3,4,5,6,9,10,11,12,13,14,15,16,17-tetradecahydro-1H-cyclopenta[a]phenanthren-3-yl (phenylsulfonyl)carbamate] (87% yield).

^1^H NMR (500 MHz, Chloroform-*d*) δ 8.04 (d, *J* = 7.7 Hz, 2H), 7.65 (q, *J* = 5.6, 3.6 Hz, 1H), 7.55 (t, *J* = 7.7 Hz, 2H), 5.18 (dd, *J* = 11.8, 7.4 Hz, 1H), 5.14 – 5.10 (m, 1H), 4.57 (tt, *J* = 10.8, 4.7 Hz, 1H), 2.45-1.10 (m, 23H), 1.00 (d, *J* = 6.6 Hz, 3H), 0.98-0.80 (m, 11H), 0.77 (s, 3H), 0.53 (s, 3H); ^13^C NMR (126 MHz, CDCl_3_) δ 149.79, 139.45, 138.56, 135.60, 133.89, 131.90, 128.93, 128.38, 126.99, 117.15, 55.90, 55.00, 49.11, 43.24, 42.79, 40.47, 39.97, 39.30, 36.61, 34.08, 33.46, 33.07, 30.93, 29.69, 29.37, 28.06, 27.26, 22.88, 22.69, 21.44, 21.10, 19.94, 19.63, 17.58, 14.12, 12.89, 12.08. HRMS (ESI-TOF) calculated for C_35_H_52_NO_4_S ([M + H]^+^): 582.3617, found: 582.3621.

**Peroxide ergosterol (Compound 6, EP).** Extracted EP [(3S,5S,8S,9R,10R,13R,14R,17R)-17-((2R,5S,E)-5,6-dimethylhept-3-en-2-yl)-10,13-dimethyl-1,3,4,9,10,11,12,13,14,15,16,17-dodecahydro-2H-5,8-epidioxycyclopenta[a]phenanthren-3-ol ] was validated by comparison with previously reported NMR studies.[[5]](#footnote-5)5 The isopropanol extraction of the mushroom powder (1 g) of *Ganoderma lucidum* was concentrated and subjected to Biotage silica gel chromatography.

^1^H NMR (500 MHz, Chloroform-*d*) δ 6.50 (d, *J* = 8.4 Hz, 1H), 6.24 (d, *J* = 8.5 Hz, 1H), 5.22 (dd, *J* = 15.3, 7.6 Hz, 1H), 5.14 (dd, *J* = 15.3, 8.3 Hz, 1H), 3.96 (dq, *J* = 11.1, 5.6, 5.0 Hz, 1H), 2.20-1.05 (m, 30H), 1.00 (dd, *J* = 6.7, 2.2 Hz, 3H), 0.91 (d, *J* = 6.8 Hz, 3H), 0.88 (s, 3H); ^13^C NMR (126 MHz, CDCl_3_) δ 135.39, 135.18, 132.28, 130.72, 82.13, 79.40, 66.45, 56.17, 51.66, 51.06, 44.54, 42.76, 39.72, 39.32, 36.94, 36.91, 34.67, 33.05, 30.10, 28.64, 23.39, 20.87, 20.62, 19.94, 19.63, 18.16, 17.55, 12.86. HRMS (ESI-TOF) calculated for C_28_H_44_N_a_O_3_ ([M + Na]^+^): 451.3188, found: 451.3191.

**Ergosterol peroxide sulfonamide (Compound 6a).** A solution of peroxide ergosterol (1.0 equiv) and benzenesulfonyl isocyanate (1.10 equiv) in dry THF was stirred at 25°C for 2 h until completion by TLC progress. The reaction mixture was then diluted with brine and extracted with EtOAc. The combined organic phase was dried over MgSO_4_ and concentrated under reduced pressure. The crude product was purified by silica gel column chromatography (Hex/EtOAc, 1:9) to provide ergosterol peroxide sulfonamide [(3S,5S,8S,9R,10R,13R,14R,17R)-17-((2R,5S,E)-5,6-dimethylhept-3-en-2-yl)-10,13-dimethyl-1,3,4,9,10,11,12,13,14,15,16,17-dodecahydro-2H-5,8-epidioxycyclopenta[a]phenanthren-3-yl (phenylsulfonyl)carbamate] (91% yield).^1^H NMR (500 MHz, Chloroform-*d*) δ 8.03 (dd, *J* = 8.3, 1.3 Hz, 2H), 7.69 – 7.62 (m, 1H), 7.55 (t, *J* = 7.8 Hz, 2H), 6.49 (d, *J* = 8.5 Hz, 1H), 6.23 – 6.14 (m, 1H), 5.21 (dd, *J* = 15.2, 7.6 Hz, 1H), 5.13 (dd, *J* = 15.3, 8.2 Hz, 1H), 4.88 – 4.80 (m, 1H), 4.22 (td, *J* = 10.5, 1.7 Hz, 1H), 4.08 (ddd, *J* = 21.1, 10.9, 2.1 Hz, 1H), 2.39 – 2.31 (m, 1H), 2.15-0.70 (m, 36H); ^13^C NMR (126 MHz, CDCl_3_) δ 149.22, 138.48, 135.12, 134.90, 134.68, 133.97, 132.32, 131.04, 130.82, 130.80, 129.16, 128.96, 128.35, 126.44, 81.60, 79.42, 73.13, 56.14, 50.78, 44.54, 42.76, 36.80, 34.03, 32.81, 30.93, 29.69, 26.05, 23.33, 20.46, 19.94, 19.62, 18.01, 17.56, 12.22, 10.81. HRMS (ESI-TOF) calculated for C_35_H_49_NNaO_6_S ([M + Na]^+^): 634.3178, found: 634.3182.

**Palmitic acid (Compound 7).**

Extracted compound **7** was validated by comparison with previously reported NMR studies[[6]](#footnote-6)6. The isopropanol extraction of the mushroom powder (1 g) of *Ganoderma lucidum* was concentrated and subjected to Biotage silica gel chromatography.

^1^H NMR (500 MHz, Chloroform-*d*) δ 2.35 (t, *J* = 7.5 Hz, 2H), 1.63 (p, *J* = 7.4 Hz, 2H), 1.40 – 1.20 (m, 24H), 0.88 (t, *J* = 6.8 Hz, 3H); ^13^C NMR (126 MHz, CDCl_3_) δ 178.41, 33.74, 31.92, 29.69, 29.68, 29.67, 29.65, 29.64, 29.59, 29.43, 29.36, 29.24, 29.06, 24.69, 22.69, 14.12. HRMS (ESI-TOF) calculated for C_16_H_32_NaO_2_ ([M + Na]^+^): 279.2300, found: 279.2303.

d) NMR data for compounds

**Figure S9.** _1_H NMR and ^13^C NMR of GA-01 (Ganoderic acid A, Acetone-d6)

**Figure S10.** _1_H NMR and ^13^C NMR of GA-01-ME (Ganoderic acid A methyl ester, CDCl_3_)

**Figure S11.** _1_H NMR and ^13^C NMR of Ergosterol (CDCl_3_).

**Figure S12.** _1_H NMR and ^13^C NMR of Ergosterol sulfonamide (CDCl_3_).

**Figure S13.** _1_H NMR and ^13^C NMR of 5,6-dehydroergosterol (CDCl_3_).

**Figure S14.** _1_H NMR and ^13^C NMR of 5,6-dehydroergosterol sulfonamide (CDCl_3_).

**Figure S15.** _1_H NMR and ^13^C NMR of Ergosterol peroxide (CDCl_3_).

**Figure S16.** _1_H NMR and ^13^C NMR of Ergosterol peroxide sulfonamide (CDCl_3_).

**Figure S17.** _1_H NMR and ^13^C NMR of Palmitic acid (CDCl_3_).

1. Qiao, Y., Zhang, X-M., Qiu, M-H. Two novel lanostane triterpenoids from *ganoderma sinense*. Molecules. 12, 2038-2046, 1985. [↑](#footnote-ref-1)
2. Kikuchi, T., Matsuda, S., Murai, Y., Ogita, Z. Ganoderic acid G, and I and Ganolicidic acid A, and B. New triterpenoids from *ganoderma lucidum*. Chem. Pharm. Bull. 33, 2628-2631, 1985. [↑](#footnote-ref-2)
3. Yang, B., Miller, P. A., Möllmann, U. & Miller, M. J. Syntheses and biological activity studies of novel sterol analogs from nitroso Diels-Alder reactions of ergosterol. *Org. Lett.* 11, 2828–2831, 2009. [↑](#footnote-ref-3)
4. ^4^ Bu, M. *et al.* Synthesis of 5α,8α-Ergosterol Peroxide 3-Carbamate Derivatives and a Fluorescent Mitochondria-Targeting Conjugate for Enhanced Anticancer Activities.*ChemMedChem* **12,** 466–474, 2017. [↑](#footnote-ref-4)
5. ^5^ Yang, B., Miller, P. A., Möllmann, U. & Miller, M. J. Syntheses and biological activity studies of novel sterol analogs from nitroso Diels-Alder reactions of ergosterol. *Org. Lett.* 11, 2828–2831, 2009. [↑](#footnote-ref-5)
6. ^6^ Knothe, G., Kenar, J. A. Determination of the fatty acid profile by ^1^H NMR spectroscopy. Eur. J. Lipid Sci. Technol. 106, 88–96, 2004. [↑](#footnote-ref-6)
